# Linking the Transcriptional Profiles and the Physiological States of *Mycobacterium tuberculosis* during an Extended Intracellular Infection

**DOI:** 10.1371/journal.ppat.1002769

**Published:** 2012-06-21

**Authors:** Kyle H. Rohde, Diogo F. T. Veiga, Shannon Caldwell, Gábor Balázsi, David G. Russell

**Affiliations:** 1 Department of Microbiology and Immunology, Cornell University, Ithaca, New York, United States of America; 2 Department of Systems Biology-Unit 950, The University of Texas MD Anderson Cancer Center, Houston, Texas, United States of America; University of Massachusetts, United States of America

## Abstract

Intracellular pathogens such as *Mycobacterium tuberculosis* have evolved strategies for coping with the pressures encountered inside host cells. The ability to coordinate global gene expression in response to environmental and internal cues is one key to their success. Prolonged survival and replication within macrophages, a key virulence trait of *M. tuberculosis*, requires dynamic adaptation to diverse and changing conditions within its phagosomal niche. However, the physiological adaptations during the different phases of this infection process remain poorly understood. To address this knowledge gap, we have developed a multi-tiered approach to define the temporal patterns of gene expression in *M. tuberculosis* in a macrophage infection model that extends from infection, through intracellular adaptation, to the establishment of a productive infection. Using a clock plasmid to measure intracellular replication and death rates over a 14-day infection and electron microscopy to define bacterial integrity, we observed an initial period of rapid replication coupled with a high death rate. This was followed by period of slowed growth and enhanced intracellular survival, leading finally to an extended period of net growth. The transcriptional profiles of *M. tuberculosis* reflect these physiological transitions as the bacterium adapts to conditions within its host cell. Finally, analysis with a Transcriptional Regulatory Network model revealed linked genetic networks whereby *M. tuberculosis* coordinates global gene expression during intracellular survival. The integration of molecular and cellular biology together with transcriptional profiling and systems analysis offers unique insights into the host-driven responses of intracellular pathogens such as *M. tuberculosis*.

## Author Summary

The impact of *Mycobacterium tuberculosis* on global health is undeniable, with ∼2 million deaths and ∼9 million new cases of tuberculosis each year. A key to the success of *M. tuberculosis* as a persistent, intracellular pathogen is its ability to survive for extended periods within professional phagocytes. Sustained growth within macrophage phagosomes requires avoiding or resisting antimicrobial mechanisms and adapting to replicate in a stressful, nutrient-restricted environment. Our understanding of the survival strategies, metabolism, and physiology of *M. tuberculosis* during intracellular growth remains incomplete. We employed multi-disciplinary approaches to gain new insights into adaptive responses that *M. tuberculosis* mobilizes to secure a productive infection. We simultaneously quantified replication and death rates, used electron microscopy to evaluate bacterial integrity, and determined the temporal changes in bacterial gene expression during a 14-day infection. By overlaying this temporal transcriptome dataset onto an extended Transcriptional Regulatory Network model, we identified regulatory pathways, stress responses, and metabolic adaptations activated during key physiological transitions over the 14 days of infection.

## Introduction

*Mycobacterium tuberculosis* (*Mtb*) has evolved successful strategies to survive in the human host and thwart the attempts of the immune system to eradicate it. The astounding morbidity and mortality caused by *Mtb* – with roughly 9 million new cases and 2 million deaths annually – is a testament to the success of this enduring pathogen. Its remarkable resistance to killing by both innate and acquired immune effectors and successful adaptation to constantly changing environments allows *Mtb* to maintain infections for decades. The ability of *Mtb* to survive and replicate within macrophages (Mφ) is central to the pathogenesis of tuberculosis [1].

A considerable body of literature has been devoted to understanding how *Mtb* survives its interaction with the Mφ [1]–[5], which serves as its niche for much of the infection cycle in the human host. The phagosome into which the bacterium is internalized fails to acquire the full complement of lysosomal characteristics due to its restricted maturation; nonetheless, this environment still represents a considerable challenge to bacterial survival. It has been suggested that *Mtb* survives better through escape into the cytosol [6], however recent work has indicated that accessing the cytoplasm leads rapidly to host cell death [7], which suggests that survival within the phagosome remains the more stable environment to which *Mtb* must adapt to succeed.

A characteristic essential for *Mtb's* survival is its ability to sense its environment and modulate genetic pathways controlling resistance mechanisms, metabolism, and other processes in a timely manner. The importance of gene regulation during intracellular survival and *in vivo* infection is illustrated by the attenuated phenotypes of numerous mutants in which key transcriptional regulators have been inactivated [8]–[11]. In previous microarray studies focusing on early stages of infection, we have characterized the initial transcriptional changes in *Mtb* immediately following Mφ invasion [12]. In the current study we focus on the long-term survival of *Mtb* within resting Mφ over the course of a 14-day infection. During this extended timeframe the bacteria appear to experience diverse and changing environments and adopt differing physiological states. Shortly after infection, the bacterial numbers decline indicating a period of acute stress as the macrophage attempts to eradicate the infection. This is followed by a second period during which the bacterial numbers remain relatively constant, presumably while the bacterium adapts to, and tries to modulate its environment. Finally, approximately 5–6 days post-infection (p.i.) the bacterial population increases, indicating that conditions to support survival and replication have been established. This model affords us unique access to unraveling *Mtb*'s adaptation strategies to the changing stresses and environmental cues experienced during the establishment of a productive infection. We have exploited a range of technical approaches to define the growth states of the bacterium through the course of this 14-day infection, and to correlate these physiological states with transcriptional profiles of the surviving bacteria to identify genetic networks consistent with survival and the establishment of a productive infection.

## Results/Discussion

### The Dynamics of Bacterial Death and Replication during Extended Macrophage Infection

The establishment of a productive infection in the macrophage is not dependent merely on the short-term response following phagocytosis but the subsequent stages of adaptation that lead to a net increase in intracellular *Mtb*. To appreciate the adaptations required by *Mtb* to enter this replicative state one has to employ a temporal approach that accurately correlates bacterial numbers, including the assessment of death versus growth, with changing transcriptional profiles. We employed three different methods to probe the growth states of *Mtb* during a 14-day intracellular infection model.

**Figure 1:**
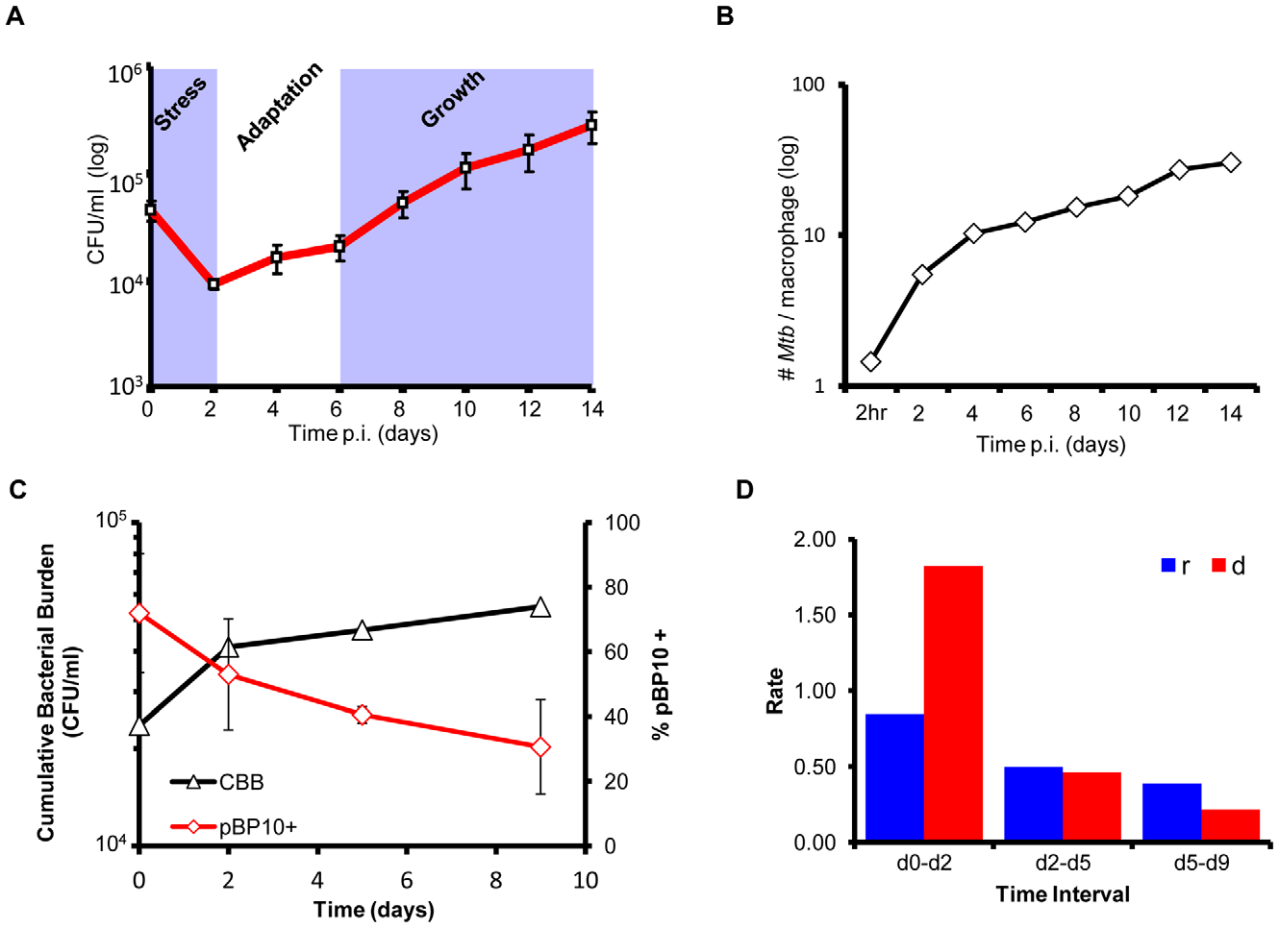
Life and death dynamics during long-term intracellular survival of *Mtb*. (A) Survival Assays. Resting murine bone-marrow derived macrophages were infected at low MOI (∼1∶1) with *Mtb* CDC1551. Viable CFU were quantified at day 0 and at 2 day intervals p.i. over a 14-day time-course by lysis of monolayers, serial dilution, and plating on 7H10 medium. Error bars indicate standard error of the mean from two independent biological replicates each consisting of three technical replicates per strain (total of 6 wells/strain). (B) Quantitative EM. The intracellular bacterial load was determined by counting the number of *Mtb* (both morphologically normal and damaged bacilli) per macrophage (100 cells counted) in samples fixed at 2-day intervals p.i. from 0–14 days. (C) Replication Clock Plasmid. The percentage of bacteria containing the pBP10 plasmid during growth in resting macrophages was determined by comparing CFU (mean ± s.d.) recovered on kanamycin vs. nonselective media (red). The cumulative bacterial burden (CBB) (black) was determined by mathematical modeling based on total viable CFU and plasmid frequency data. Data shown represents two independent experiments with each sample performed in quadruplicate (8 total wells/time point). (D) Calculated growth (blue) and death (red) rates during phases of *Mtb* intracellular survival (per day during indicated intervals).

First, resting primary Mφ isolated from C57BL/6 mice were infected with *Mtb* at a low multiplicity of infection (MOI) of 1∶1. At two-day intervals, infected monolayers were lysed and dilutions plated to determine viable colony forming units (CFU). Supernatants were also diluted and plated (prior to lysis of Mφ) to monitor extracellular bacilli that could contribute to reinfection. An initial 0.5 log decrease in cell-associated viable CFU at day 2 was followed by a period of minimal change (days 2–6) and then a more steady increase in CFU (days 6–14) (Figure 1A). This profile, consistent with our previous observations [13], [14], suggested an early killing phase followed by delayed adaptation of surviving organisms to survive and replicate within Mφ phagosomes. At every time point, extracellular *Mtb* totaled less than 5% of the intracellular burden (data not shown) suggesting that re-infection is minimal.

The intramacrophage growth of *Mtb* was also monitored by parallel transmission electron microscopy (TEM) analysis of fixed infected Mφ samples (Figure 1B). Enumeration of both morphologically normal and damaged *Mtb* in 100 randomly chosen macrophages revealed a rapid increase in the number of detectable bacteria per cell from 1.5 *Mtb*/cell at 2 hr post-infection (p.i.) to 10.3 *Mtb*/cell by day 4 (∼1.7-fold increase/day). From day 4 to day 14, the average bacterial burden increased at a notably slower rate (∼0.3-fold increase/day) (Figure 1B, Figure S1A). Interestingly, this early period of rapid *Mtb* replication following Mφ invasion coincided with the marked decrease in viable CFU (Figure 1A). This indicated that early in the infection bacterial replication is rapid, but is countered by effective bacterial killing by the Mφ.

To further validate this conclusion, we calculated *Mtb* growth and death rates during long-term infection of resting Mφ based on the loss of an unstable replication clock plasmid [15]. The proportion of intracellular *Mtb* retaining the clock plasmid pBP10 was determined by plating bacteria on media with or without 25 µg/ml kanamycin. The profile of pBP10 plasmid loss (Figure 1C, red) indicated an initial period of rapid replication (day 0–day 2) followed by an extended phase of much slower cell division (day 2–day 9). The cumulative bacterial burden (CBB) – the total number of *Mtb* live, dead, or degraded within Mφ during the infection – was calculated based on the mathematical model developed by Gill *et al.* [15]. The predicted CBB (Figure 1C, black) at day 2 p.i., closely mirrored the quantitative TEM results enumerating all detectable bacteria regardless of viability, at ∼12-fold higher than the number of viable CFU determined by plating. As shown in Figure 1D, this indicates substantial replication coinciding with even greater death rates during the early phase of infection. However, following the adoption of a slower growth rate from day 2 of infection, *Mtb* exhibited a steady increase in viable CFU.

These data indicate that, following invasion of Mφ, *Mtb* encounters a bottleneck during which the rate of bacterial killing outpaces its relatively rapid rate of replication. A similar early phase of pronounced bacterial killing was noted *in vivo* by Gill *et al.* during the first 14 days in an *Mtb*-mouse model of infection [15]. Following day 2, a period of apparent adaptation ensues extending to approximately day 6, during which both the rate of bacterial replication and the rate of killing decrease. The subsequent overall increase in viable CFU reflects further enhanced survival and the successful establishment of a productive infection. The shift in the balance between replication and death over time (Figure 1D) suggests that the successful adaptation of some *Mtb* cells to avoid killing by Mφ-derived effectors is at least as important as mechanisms for sustained replication within phagosomes. It is tempting to speculate that the slower growth rate of *Mtb* at later time points may contribute to the shift in growth∶death balance, perhaps by rendering *Mtb* more resistant to Mφ-derived pressures. Studies modeling the dynamics of *Mtb*-host interactions within human granulomas by Segovia-Juarez *et al.* support this idea, showing that slow intracellular growth rates are correlative with Mtb survival [16]. This novel insight into the life∶death equilibrium of *Mtb* during a sustained model of infection provides a physiological context for the interpretation of the global gene expression profiles discussed hereafter.

### EM Analysis Reveals Considerable Heterogeneity in the Bacterial Population during Adaptation

Descriptions of host-pathogen interactions based on population-level data, such as microarrays, are often interpreted without appreciation of the heterogeneity within that population. With this in mind, we performed detailed TEM image analysis of *Mtb*-infected Mφ at 2 day intervals over 14 days simultaneously with survival assays (Figure 1) and microarrays (see below).

**Figure 2:**
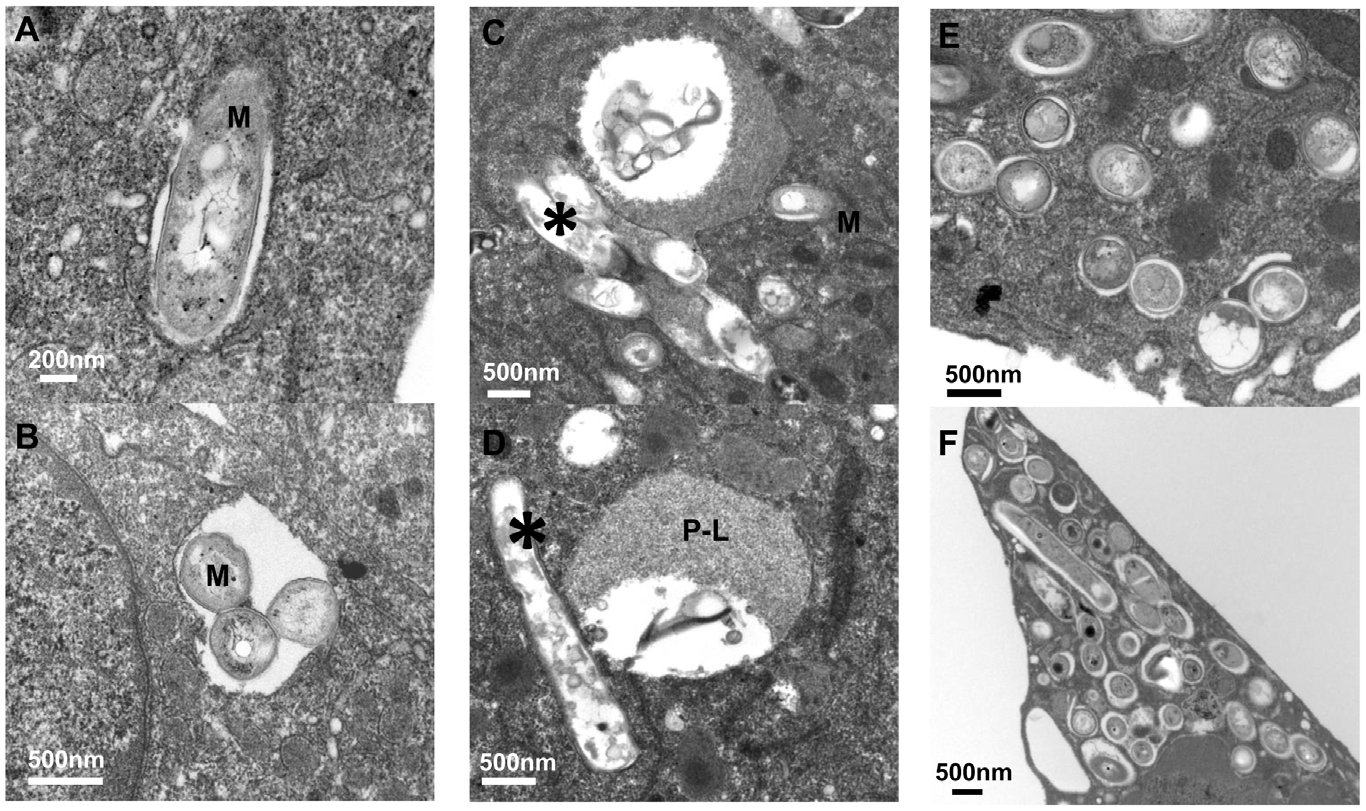
Electron microscopy analysis of long-term *Mtb*-macrophage interactions. (A–B) Intracellular *Mtb* (indicated by “M”) 2 hr p.i., with individual bacilli occupying typical tight phagosomes (A) and clusters contained in loosely apposed spacious vacuoles (B). (C–D) Damaged and degraded *Mtb* (asterisk) associated with phagolysosomes (P-L) at 2 days p.i. (E–F) Macrophages with heavy bacterial burden at day 6 (E) and day 8 (F) p.i.

**Figure 3:**
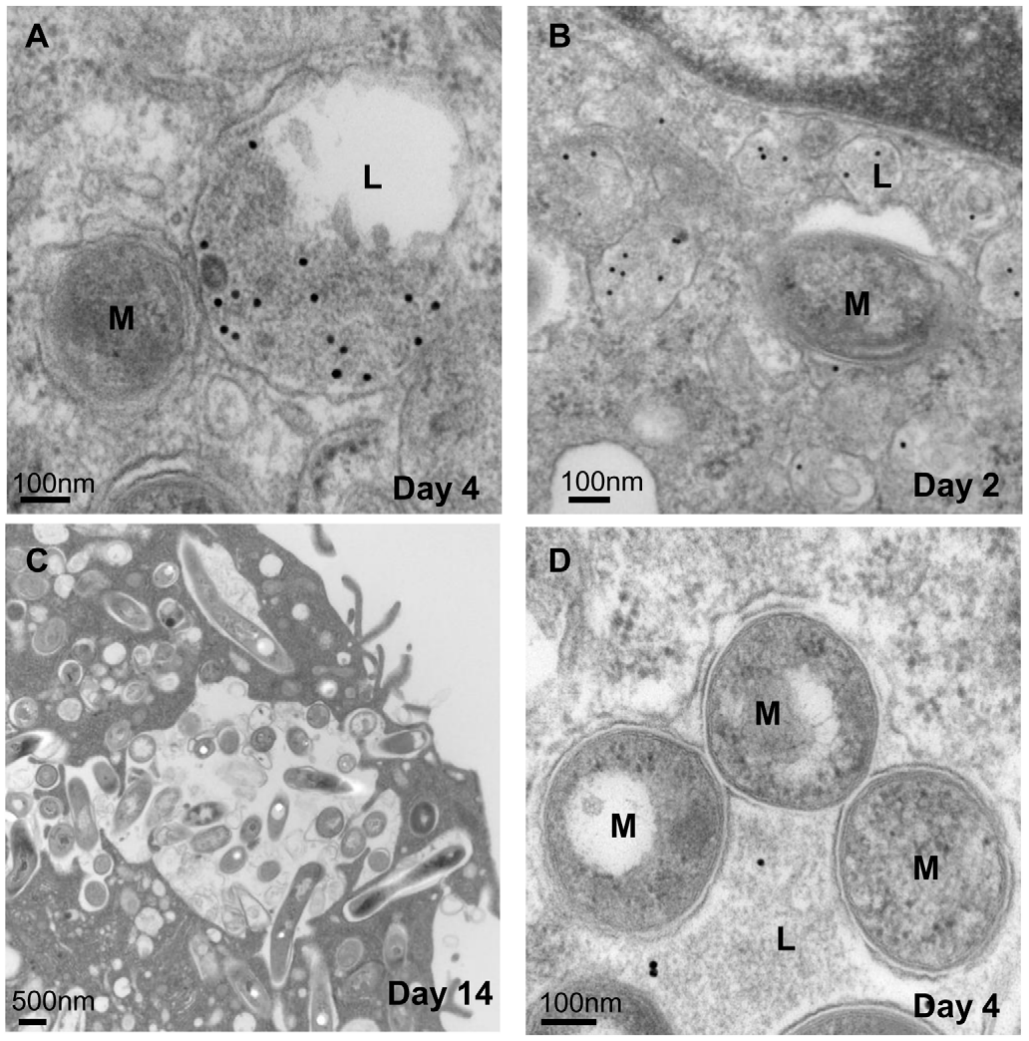
*Mtb* occupies heterogeneous intracellular niches. (A) *Mtb* in tightly apposed phagosome resisting fusion with lysosome (L) loaded with colloidal gold (day 4 p.i.). (B) *Mtb* vacuole in the process of fusion with gold-containing lysosomes (day 2 p.i.). (C) Mixture of intact and damaged/degraded *Mtb* in single large lysosomes (day 14 p.i.). (D) Cluster of morphologically intact bacilli in gold-loaded lysosomes (day 4 p.i.).

Consistent with previous observations [17]–[20], at 2 hours p.i. most single bacteria resided in a vacuole surrounded by a phagosomal membrane tightly apposed to the *Mtb* cell wall (Figure 2A). While intracellular *Mtb* proved to be quite effective at resisting fusion with lysosomes, identified following endocytic uptake of colloidal gold (Figure 3A), ∼25% of single *Mtb* did traffic to phagolysosomes (P-L) and colocalize with colloidal gold as early as 2 hr p.i. (Figure 3B). At 2 days p.i., image analysis of *Mtb*-infected Mφ revealed an increased frequency of morphologically-damaged *Mtb* in large granular lysosomes (Figure 2C–D), consistent with the early phase of killing of replicating *Mtb* indicated by the clock plasmid experiments. Whereas 99% of bacteria in the inoculum cultures were scored as intact, by day 2 only 75% of phagocytosed *Mtb* appeared morphologically normal. We also noted heavily damaged *Mtb* that appeared as hollowed out “ghosts”, but these were not scored as damaged. The discrepancy between the magnitude of killing, calculated based on clock plasmid data (Figure 1D), and the small proportion of visibly damaged bacteria as well as the constant ratio of normal to damaged bacilli (Figure S1A) suggests a relatively efficient clearance of nonviable organisms. At later time points, the bacterial population continued to increase (Figure 2E–F, Figure S1B) with up to ∼150 *Mtb*/Mφ. In contrast to a recent report that a large subset of *Mtb* H37Rv translocated into the cytosol of Mφ within 48 hrs [6], phagosomal membranes were definitively detected surrounding ∼90% (88% at day 4 p.i., 86.5% at day 8 p.i.) of intracellular *Mtb* CDC1551 throughout the duration of the infection (25 Mφ and >100 *Mtb* examined per time point).

There was a surprising degree of heterogeneity of compartments in which visibly intact *Mtb* appeared to reside during long-term infections of Mφ, including “replicating” *Mtb* in typical tight niches, small fused P-L, and putative double membrane-bound autophagosomes that colocalized with gold (Figure 3, Figure S2). The diversity of intra-host environments undoubtedly contributes to heterogeneity within the bacterial population.

### *Mtb* Transcriptional Adaptation during Long-term Macrophage Infection

**Figure 4:**
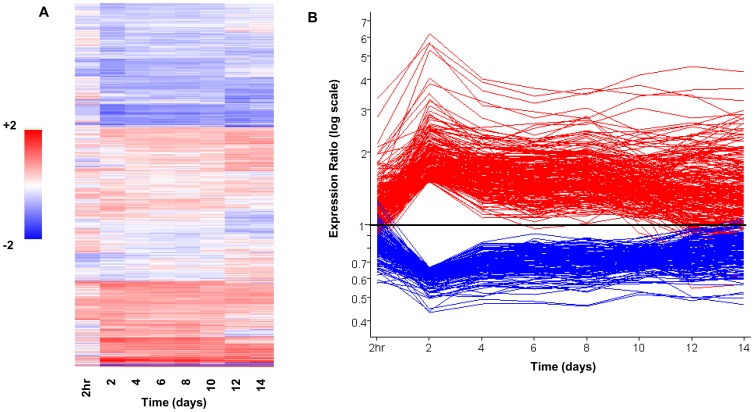
Dynamic *Mtb* transcriptome during long-term macrophage infection. (A) Gene tree of intracellular gene expression relative to 2 hr “no macrophage” control CDC1551 (two biological replicates of complete time-course). Expression profiles for 3626 genes passing quality filters (flagged as present in 14 of 16 arrays) and showing significant changes in gene expression were clustered using the Euclidean distance algorithm. Significantly regulated genes were defined by combining static (p<0.05 in at least one time point) and dynamic measures (q<0.03 by EDGE analysis). Each column represents the global transcription profile at the designated time p.i.. Red and blue indicate higher or lower gene expression than the control, respectively. Unless otherwise indicated, the color scale for expression (2-fold up or down) was used for all subsequent figures. (B) The “bottleneck” response. Temporal expression profiles of genes differentially regulated at Day 2 p.i., including genes from (A) that were up- (red) or down-regulated (blue) >1.5-fold (shown as ratio of signal intensity relative to control). Note the maximal change in transcript levels at day 2 p.i. followed by majority trending back towards control levels.

The transcriptional profile of *Mtb* during the course of the 14 day infection should mirror the physiological states and transitions defined in the previous section. Therefore, overlaying the temporal transcriptional changes with these physiological states will generate a correlative linkage between the two datasets. We conducted microarray analysis of RNA isolated from intracellular *Mtb* at 2 hr p.i. and at 2 day intervals over a 14 day period (GEO accession GSE35362). Fluorescent amplified RNA (aRNA) targets from each time-point were prepared from linearly amplified total *Mtb* RNA and hybridized against a 2 hr “no Mφ” control as previously described [12], [13]. This enabled us to determine dynamic expression ratios over time relative to a single common denominator mRNA sample, in this case an uninfected control time-matched with the earliest time-point (2 hr). Semi-quantitative RT-PCR of select target genes (*aprAB, hspX, bfrB, icl, groEL2, katG, whiB7*) was conducted to confirm temporal array profiles ([21] and data not shown). We identified 3626 genes with significant changes in expression during extended Mφ infection by combining both static statistical cutoffs (p<0.05 in at least one time-point) and EDGE (*E*xtraction and analysis of *D*ifferential *G*ene *E*xpression) methodology (Figure 4A) [22]. EDGE analysis identifies temporal changes in transcript levels that would not be deemed statistically significant at any single time-point by standard p-value measurements.

### Temporal Gene Expression Profiles during Intracellular Adaptation

Our data indicate that *Mtb* encounters a bottleneck during the first two days p.i. during which a subpopulation is killed before surviving bacilli enter a sustained period of enhanced survival, albeit at a reduced replication rate. Temporal dynamics of the *Mtb* transcriptome during intracellular adaptation highlight genes responding, and thus likely active, during specific stages of infection. Given the short half-life of bacterial mRNA [23], [24] and steady clearance of nonviable *Mtb*, the transcriptional profiles observed are likely derived only from the surviving bacilli.

#### Up to day 2: Surviving the day 2 bottleneck

The transcriptome of bacilli sampled every 2 days largely reflected this transition, with gene expression changes of maximal magnitude and complexity occurring at day 2 (Figure 4B). We propose that most of these genes are regulated in immediate response to the stressful conditions encountered upon invasion of the macrophage. The continuity of the expression profiles over time beyond this early crisis phase argues that these are specific adaptive transcriptional trends. Transcript levels of a substantial portion of this geneset then gradually declined over the next 12 days (Figure 4B, Table S1) including genes involved in general stress responses (*hsp, dnaK, groEL2*), stress response regulation (*sigB*), anaerobic respiration (type II NADH dehydrogenase, *ndh2*), Fe-S cluster assembly (*Rv1460, 1461, 1465*), regulation of acetate and glyoxylate cycle metabolism (*Rv0465c*) [25], propionate metabolism *(Rv1129c, prpC (Rv1131), prpD (Rv1130))*, and the starvation response (*lat*). This temporal profile indicates that cues driving these responses are likely most acute as the bacteria try to maintain rapid early intracellular growth amidst an array of host-derived pressures.

Schnappinger *et al.* previously reported the intra Mφ gene expression of *Mtb* strain 1254 over a short-term infection out to 2 days p.i. [26]. The identification of 88 genes that were reliably detected and induced >1.5-fold in both studies at day 2 confirms key transcriptional responses of *Mtb* to the phagosomal environment, the majority of which remain elevated for the duration of the 14-day infection. These included genes known to be involved in general stress responses (*hsp, dnaK, clpB*), carbon metabolism (*pckA, icl, prpC, prpD*), oxidative stress (*Rv1461, 1463*), iron storage (*bfrB*), hypoxia and nitrosative stress (*Rv1738, Rv2626c*), and secreted antigens (*mpt70, mpt83*). This consistency with previously reported datasets from short-term infection models argues the general validity of the approach and the significance of the data from later time points in our extended infection model.

We also detected 137 additional genes upregulated at day 2 that were specific to this study including 17 members of the PhoPR regulon [27] (Figure S3A, Table S2) as well as numerous “MT genes”, comprised of 371 ORFs predicted in a later annotation of the *Mtb* CDC1551 genome whose intracellular expression has not been reported previously (Figure S3B, Table S3). The differential expression of MT genes during long-term Mφ infection suggests they may play important roles in adaptation to the phagosomal environment. This hypothesis is substantiated by our recent characterization of the MT2466-MT2467-Rv2396 locus (renamed *aprABC*), which showed rapid induction upon acid stress or Mφ invasion and is known to regulate carbon flux into cell wall lipids to support intracellular survival [21].

**Figure 5:**
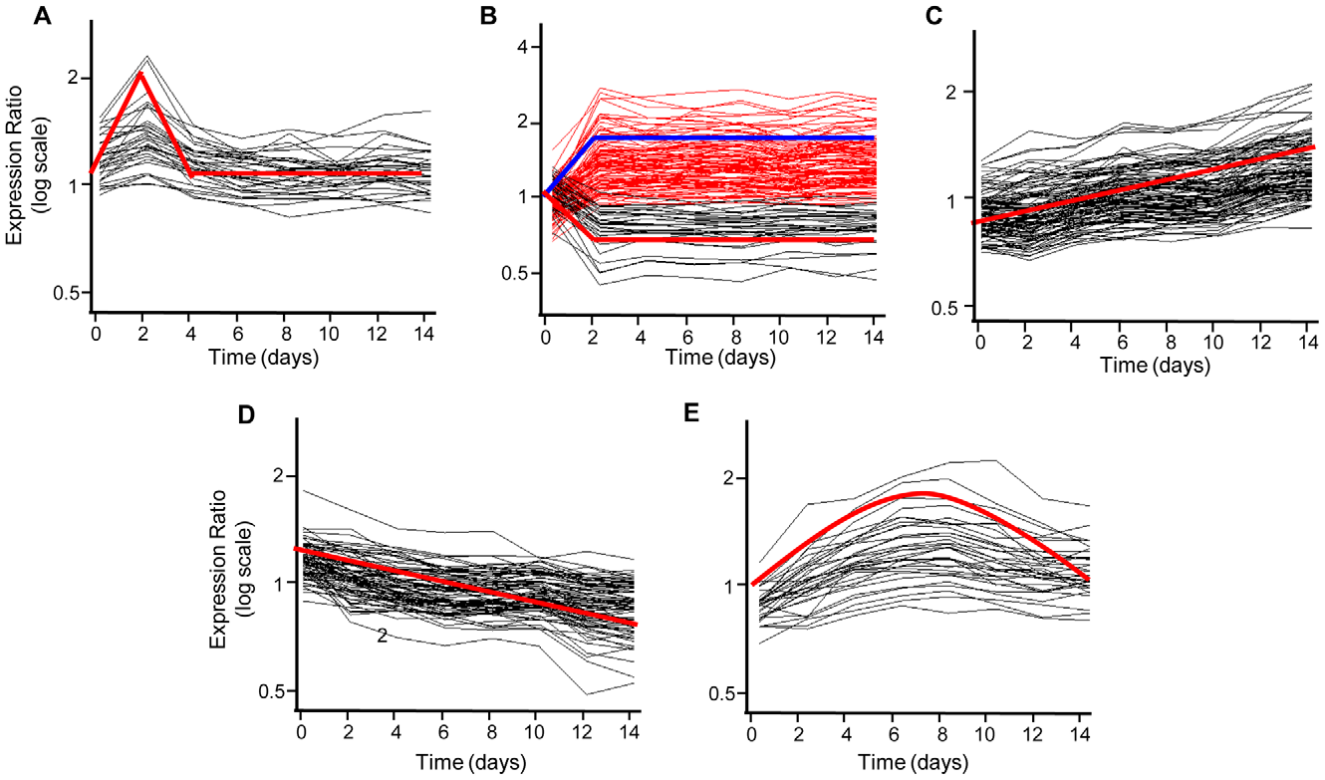
Profiles of Coordinated Temporal Gene Regulation. (A–E) Clusters of temporally co-regulated genes were identified by correlation (>0.9) with synthetic profiles, indicated by red (A,B (down), C–E) or blue (B, up) lines. (A) Genes transiently induced at day 2, followed by return to control levels. B) Early induced (red) and repressed (black) genes with sustained altered transcript levels. Genes with steadily increasing (C) or decreased (D) expression levels. (E) Delayed induced genes.

The transient induction of a geneset including *whiB7* and adjacent ORF *MT3290.2* at Day 2 followed by a return to baseline levels (Figure 5A, Table S4) reveals genes active specifically during this early stage of infection. WhiB7, a member of an Actinomycete-specific family of Fe-S cluster proteins, mediates enhanced antibiotic resistance by upregulation of a defined regulon in response to antibiotics and fatty acids [28]. The role of *whiB7* during intracellular survival remains to be elucidated. It appears that as *Mtb* replication slows and the bacterium adapts to the phagosomal environment, many stressors and corresponding transcriptional responses return to control levels.

#### Day 2 onwards: Survival and growth beyond the Bottleneck

The temporal changes in *Mtb* gene expression during the intracellular adaptation phase (following day 2) will indicate the metabolic pathways and survival mechanisms required for establishment of an environment supportive to bacterial growth. For example, Figure 5B (shown in red) shows 103 genes induced early (maximal by day 2 p.i.) that remain elevated throughout the 14-day timecourse (Table S5). This subset includes genes involved in fatty acid metabolism (*pckA, icl, ald, Rv0448c-0449c*), KstR-dependent cholesterol metabolism (*igr*), secreted antigens (*mpt83-Rv2874-mpt70*), and regulators (*Rv2359*). In a previous study, we characterized the functional impact of genetic diversity on *Mtb*-host interactions and gene expression by analyzing the intracellular transcriptome profiles of a globally diverse panel of *M. tuberculosis* complex (MTC) clinical isolates [13]. We discovered a set of genes conserved and similarly regulated within Mφ phagosomes across all MTC clinical isolates at 24 hr p.i. which we termed the “core transcriptome”. While the induction magnitudes and kinetics varied, the majority of the 215 genes belonging to the resting Mφ core transcriptome also exhibited early, sustained induction profiles (Figure S4, Table S6). The conserved nature of this response across bacterial isolates and its sustained expression throughout the duration of the infection implies strongly that these genes are important for maintaining infection within Mφ.

In contrast, looking at genes with a reciprocal pattern of sustained down-regulation, we found ∼30 genes (shown in black) with a profile of early, sustained repression including numerous ribosomal genes (*rplF*, *rplI*, *rplJ*, *rplV*) (Figure 5B, Table S7). This correlates well with the decreased replication rate revealed in the clock plasmid assay. Gene subsets were also identified that exhibited increasing (103 genes) or decreasing (78 genes) transcript levels throughout (Figure 5C, Table S8 and Figure 5D, Table S9 respectively), indicative of responses to stimuli that steadily intensified or diminished during the infection. 

Although transcript levels for the vast majority of genes were altered by day 2, we did identify a set of genes that exhibited a delayed “bell-curve” pattern of expression (Figure 5E, Table S10). This profile suggests that *Mtb* may encounter novel host-derived pressures at later time points or that prolonged growth within the phagosomal environment requires additional bacterial adaptation. This genelist included several transcriptional regulators (*Rv2989, Rv2516c*) and fatty acid metabolism genes (*fadE9, mmsA, Rv0762c, Rv0764c-0765c*). In contrast to early responses likely triggered by immediate stress, *Mtb* may also encounter more insidious pressures linked to evolving metabolic requirements.

### Temporal Expression of Signature Regulons during the Infection

In addition to the temporal alterations in the transcriptional profile, we also examined transcription profiles of genesets linked to some of the more well-characterized environmental responses to probe how they matched the physiological transitions we have proposed occur during the 14-day infection. We have focused on the DosR regulon, regulation of carbon flux, and the response to pH, as some of the better characterized physiological themes.

#### Expression of the DosR dormancy regulon

**Figure 6:**
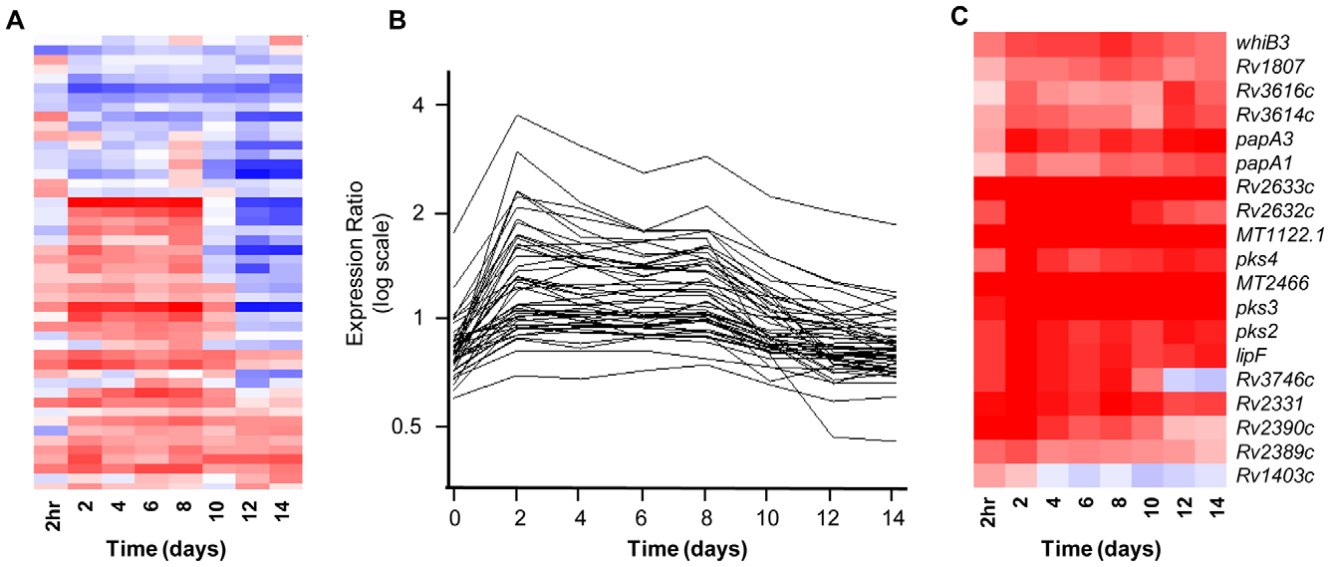
The transcriptome as a bioprobe of the *Mtb* intracellular environment. Gene trees of select signature genesets that are useful as surrogate readouts of the conditions encountered by *Mtb* within macrophages. (A) Hypoxia/RNI/CO. Genes of the well-characterized *dosRS*-dependent “dormancy” regulon [29] indicate exposure of *Mtb* to known cues of this geneset (hypoxia, reactive nitrogen intermediates (RNI), and carbon monoxide (CO)). (B) “Guilt by association” analysis. Genes regulated in synch with known virulence regulons – i.e. the DosR regulon - were identified by using a highly regulated member of this regulon, *hspX*, in place of synthetic profiles. (C) Acid Stress. Select members of a regulon activated by acidic pH *in vitro* and within macrophage phagosomes [12] report on the pH encountered by *Mtb* within its vacuole.

Figure 6A depicts the expression profiles of the so-called “dormancy” regulon, a set of ∼50 genes induced by the DosRS/T two-component system in response to multiple signals including hypoxia, nitrosative stress, and carbon monoxide [29]–[32]. The DosR regulon appears to play a role during transitions into and out of stress-induced dormant states [33]. Although DosR induction is typically associated with hypoxic conditions within granulomas and reactive nitrogen intermediates (RNI) derived from activated Mφ, we noted induction of ∼60% of DosR-dependent genes in resting Mφ within 48 hr p.i., with upregulation of some transcripts detectable as early as 2 hr p.i. (Figure 6A). A subset of DosR-dependent genes triggered by hypoxia *in vitro* remained unchanged in resting Mφ phagosomes, perhaps reflecting distinct threshold levels of sensitivity to inducing signals. It is conceivable that all three known cues of *DosRS/T* signaling are acting in concert within the resting phagosome. Greiss assay analysis of supernatants from 14-day infected Mφ showed a spike in nitrate levels at day 2 p.i. that rapidly declined to baseline levels by day 4 (data not shown), implicating nitric oxide as a signal of early DosR regulon induction. Decreased intraphagosomal oxygen concentrations in macrophages [34] and/or carbon monoxide generated by infection-induced heme oxygenase-1 (HO-1) may influence the expression of DosR-dependent genes. Finally, the expression of DosR-dependent genes may be modulated by slow growth rates and nutrient limitation during intraphagosomal growth [35].

The most striking aspect of dormancy gene expression during long-term Mφ infection was the sudden repression of DosR-dependent genes at ∼day 8, despite steady transcript levels of *dosR*, *dosS*, and *dosT* (Figure 6A). The downregulation to sub-control levels of some DosR-dependent genes that were not significantly induced at earlier time points suggests active repression, as well as an intricate crosstalk between DosR and other transcriptional regulators to coordinate this stress response. Taking a “guilt-by association” approach, we utilized the temporal profile of *hspX*, a member of the DosRS-dependent dormancy regulon [29], to identify ∼50 additional early-induced genes (Figure 6B, Table S11) that exhibited the striking down-regulation at ∼Day 8 noted for the DosRS regulon (see below). The coordinated regulation of poorly characterized genes with known regulons or stimulons offers a starting point for elucidating their function. We suggest that this is a highly significant transcriptional pattern because it corresponds to the time point where *Mtb* transitions from a period of adaptation to net intracellular growth.

This shift in *Mtb*-Mφ interactions may reflect the sudden diminution of the cue(s) driving the response. Alternatively, this profile may reflect the staged adaptation of *Mtb* to phagosomal stresses such as hypoxia, NO, and/or CO. Balazsi *et al.* reported similar induction of the DosRS regulon followed by a downward shift at ∼day 8 in an *in vitro* hypoxia model [36]. This profile is reminiscent of the enduring hypoxic response (EHR) described by Rustad *et al.* (Figure S5) in which *DosRS* is only active during the early phase of extended exposure to hypoxia [37]. Although unable to cope with rapid shifts to hypoxic conditions, *Mtb* can survive gradual oxygen depletion by coordinated reprogramming of gene expression and metabolism [38]–[40]. Early exposure to *dosRS*-inducing conditions within resting Mφ may allow *Mtb* to pre-adapt for harsher conditions within activated Mφ and hypoxic granulomas.

#### Adaptation of carbon metabolism during extended intracellular growth

**Figure 7:**
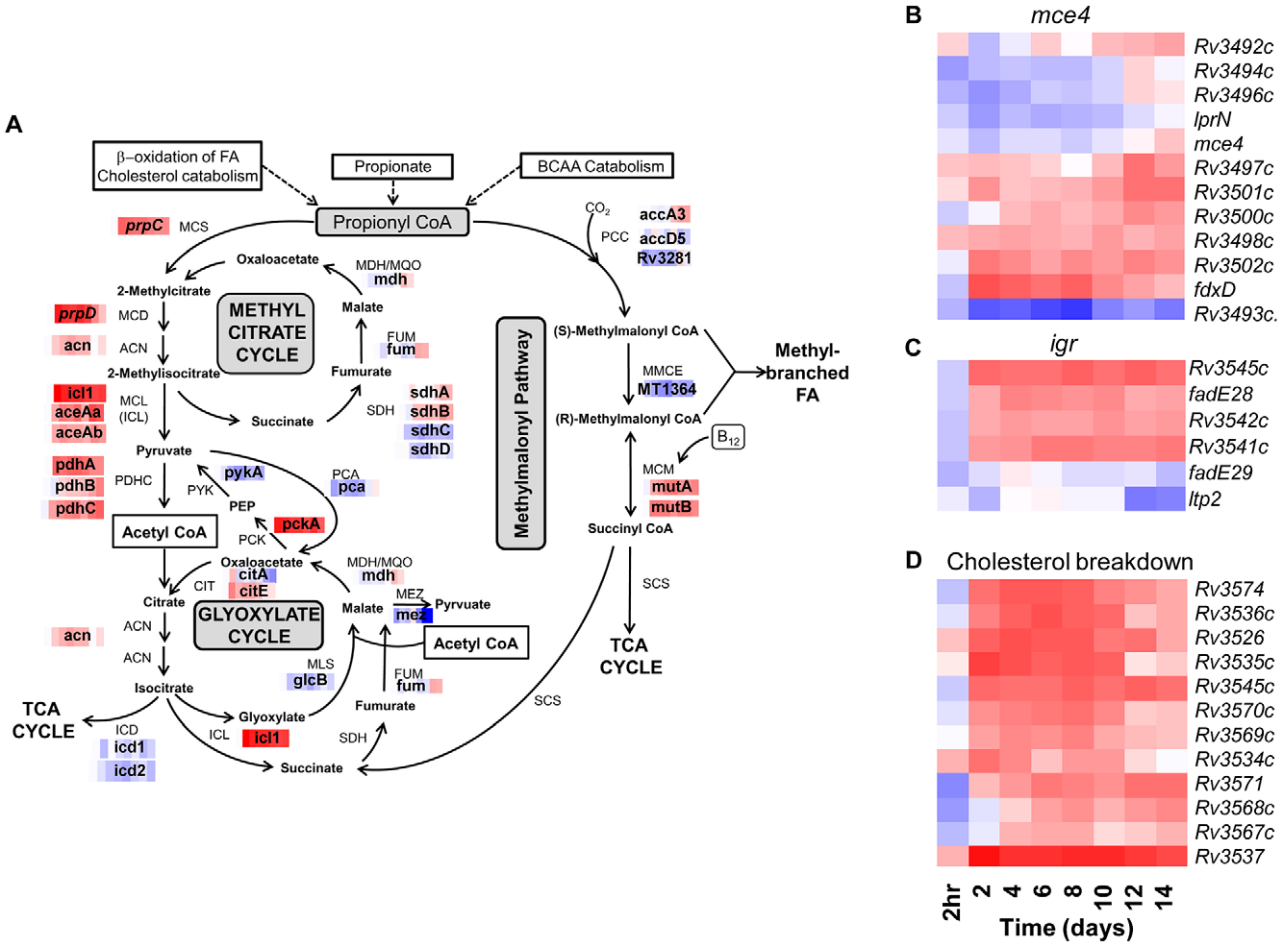
Adaptation of Carbon Metabolism during Extended Intracellular Growth. Gene trees showing phagosomal induction of loci implicated in utilization of host-derived cholesterol as a carbon source. (A) Expression data from *Mtb* during a 14-day macrophage infection were superimposed onto a pathway map depicting propionyl-CoA metabolism adapted from Savvi *et al.* [54]. The concerted induction of the methylcitrate cycle indicates activation of this pathway for detoxifying by-products of propionyl-CoA metabolism during macrophage survival. (B) *mce4* is involved in the uptake of exogenous cholesterol [47], [49]. The *igr* locus (C) and genes of the *kstR* regulon (D) are required for metabolism of cholesterol [46], [51].

Sequestration of *Mtb* within Mφ phagosomes undoubtedly imposes limitations on the nutrients available for growth. Our temporal gene expression analysis revealed that realignment of carbon metabolism pathways to allow utilization of host-derived fatty acids (FA) and cholesterol is a key component of *Mtb* intracellular adaptation. We noted early, sustained induction of *pckA* (phosphoenolpyruvate carboxykinase) and concomitant repression of *pca* (pyruvate carboxylase) (Figure 7A). *pckA* is induced by fatty acids, upregulated during mouse infection [41] and required for mycobacterial survival in both Mφ and mice [42], [43], highlighting the importance *in vivo* of gluconeogenesis of carbon derived from fatty acid β-oxidation. The rapid induction of *icl*, encoding the isocitrate lyase component of the glyoxylate shunt [44], is consistent with the availability of fatty acid carbon sources within resting phagosomes. The lack of induction of the second gene of the glyoxylate shunt, *glcB* (malate synthase) (Figure 7A), suggests an alternate role for *icl* (discussed below). Previous studies have demonstrated the ability of *Mtb* to utilize cholesterol as a source of energy and carbon [45]–[48]. The activation of the majority of the KstR regulon by day 2 p.i., including the *mce4* [47], [49] and *igr* [46], [50] loci, indicates that *Mtb* likely has ready access to cholesterol within resting Mφ (Figure 7B–D, Figure S6). KstR is known to be induced by cholesterol [51] and multiple KstR-dependent genes have been implicated in cholesterol uptake and metabolism [45]–[49], [52], [53]. In particular, the *mce4* locus encodes a dedicated transport system capable of internalizing cholesterol [47], [49]. Note that a subset of genes predicted to be directly controlled by KstR exhibit unique expression patterns (lack of induction) during extended Mφ infection (Figure S6D). This could be due to inaccurate identification of KstR-controlled genes or, more likely, additional layers of regulatory input acting within the vacuole.

A potentially deleterious side-effect of cholesterol catabolism and β-oxidation of odd-chain length fatty acids is the generation of toxic metabolites including propionyl-CoA and downstream intermediates [46], [54], [55]. As shown in Figure 7A, *Mtb* is equipped with three pathways to detoxify and utilize propionyl-CoA as either a carbon source or building block for methyl-branched fatty acid components of the cell wall: methylcitrate cycle (MCC), methylmalonyl pathway (MM), and synthesis of methyl-branched fatty acids. To identify propionyl-CoA detoxification mechanisms activated during long-term intracellular survival, we overlayed temporal expression profiles of relevant metabolic genes (shown as heat maps) onto the pathway map (Figure 7A, adapted from Savvi *et al.* [54]). The concerted induction of *icl1* and *prpCD* reflects enhanced activity of the MCC within Mφ, serving to convert propionyl-CoA into pyruvate and succinate. In addition to its role in the glyoxylate shunt pathway as an isocitrate lyase (ICL), the bifunctional *icl1* is also a 2-methylisocitrate lyase (MCL) that interacts with *prpC* (encoding methylcitrate synthase) and *prpD* (encoding methylcitrate dehydratase) in the MCC [56]. Mutants lacking *icl1*, *prpC*, and/or *prpD* were unable to grow on propionate as a sole carbon source. In contrast to the normal growth of Δ*prpCD Mtb in vivo*, a Δ*icl1* strain was markedly attenuated in mice [55], [56]. These paradoxical phenotypes are consistent with a model invoking accumulation of *prpCD*-generated toxic propionyl-CoA metabolites in the absence of MCL activity rather than the inability to utilize fatty acids due to interruption of the glyoxylate shunt. In a recent study, Griffin *et al.* confirmed the important role for the MCC enzymes *prpCD* regulated via Rv1129c for cholesterol utilization during intracellular survival [57].

Other than a small increase in *mutAB* (methylmalonyl-CoA mutase) transcript levels, we did not detect coordinated upregulation of the MM pathway during *Mtb* infection of resting Mφ. This may be due to inactivation of the MM pathway in our infection model due to the absence of vitamin B_12_, a cofactor required for MutAB activity [54]. Derivatives of propionyl-CoA can also be diverted into the biosynthesis of methyl-branched fatty acids such as phthiocerol dimycocerosate (PDIM), sulfolipid (SL), and di-and poly-acyl trehaloses (DAT, PAT). Whereas PDIM and mycolic acid synthesis appeared downregulated by intracellular *Mtb* (Figure S7), we noted marked induction of *pks2-papA1* (SL) and *pks3-pks3-papA3-mmpL10* (DAT, PAT) upon internalization of *Mtb* by Mφ. We have previously linked the rapid activation of these loci within minutes of phagocytosis to acidification of the vacuole [12]. Thus, enhanced synthesis of SL, DAT, and PAT may serve as a sink to prevent accumulation of toxic byproducts of intracellular fatty acid and cholesterol metabolism.

#### The Acid Response regulon

In a previous study, we demonstrated that the drop in pH experienced in transition from the extracellular milieu to ∼pH 6.4 in the phagosome of a resting Mφ serves as a dominant signal to *Mtb* for early intracellular gene induction [12]. Genes of this acid regulon exhibited rapid and sustained induction throughout long-term Mφ infection (Figure 6C). Thus, acid pH serves as an early cue of Mφ invasion as well as a protracted stressor during the period of slowed replication. The specific roles of acid-induced genes in directly mediating pH homeostasis and/or intracellular survival are becoming clearer. Remodeling of the cell wall in response to phagosome acidification, indicated by the induction of *pks2-papA1* (sulfolipid (SL) biosynthesis) and *pks3-pks4-papA3* (di- and poly-acyltrehalose (DAT, PAT) biosynthesis) (Figure 6C), suggests one potential mechanism of coping with this intracellular stressor. The concomitant repression of genes involved in phthiocerol dimycocerosate (PDIM) during Mφ infection (Figure S7B) further validates the finding by Jain *et al.* [58] that SL and PDIM production are controlled by the availability of the common methyl malonyl CoA (MMCoA) precursor, and are linked to the bacterium's desire to regulate the propionyl CoA pool and limit its potential toxicity, as detailed in the previous section.

WhiB3, an Actinomycete-specific protein shown to modulate the incorporation of propionate into virulence lipids including SL and PDIM in response to redox signals [59], was also induced throughout the time-course (Figure 6C). The responsiveness of *whiB3* to phagosomal acidification [12], [60] suggests a link between acid stress, internal redox balance, and virulence lipid biosynthesis. The acid responsive locus *aprABC* (MT2466-MT2467-Rv2396), which exhibits early sustained induction, mediates intracellular survival via unknown mechanisms by influencing gene expression and lipid metabolism [21]. Finally, *Mtb* may rely on phagosomal acidification as a cue of Mφ invasion to coordinate the timely expression of intracellular virulence mechanisms. For example, acid induction of *espABC* (Rv3614c–Rv3616c), a locus required for ESX-1 mediated secretion of virulence factors [61], [62], may facilitate the rapid secretion of preformed Type VII effectors into the phagosome (Figure 6C, Figure S8B). We also noted the induction of ESX-1 components encoded within the RD1 locus including EspR (Rv3849), a secreted regulator of *espABC* (Figure S8) [62].

### Transcriptional Regulatory Network Response to Phagosome Cues

In addition to analyzing the transcriptional profiles according to temporal dynamics and known functional themes we also conducted a systems-level analysis to characterize the behavior of the *Mtb* Transcriptional Regulatory Network (TRN) underlying pathogen survival during the 14-day infection. This network-based approach incorporates extensive *a priori* information on *Mtb* gene regulation and network topology, combined with expression data, to assess network responses in surviving bacteria elicited by the Mφ.

We started by expanding a large-scale *Mtb* TRN containing gene regulatory interactions extracted from both experimental and computational datasets [37]. The previous TRN comprised 738 genes (18% of the genome) and 937 regulatory links obtained from three sources: (*i*) literature mining; (*ii*) *Mtb*RegList database [63]; (*iii*) inference from orthology with *Escherichia coli* [64]. To obtain the expanded network used in this study, we collected gene regulatory data from the following additional sources. (*iv*) We added orthology-based interactions inferred from the closely related *Corynebacterium glutamicum* available in the MycoRegNet database, which considerably expanded the TRN by adding 425 new interactions (Figure S9A). The extensive overlap with regulatory links supported by experimental data validated these interactions identified from orthologous Transcription Factor (TF)-Target Gene (TG) pairs in the two organisms (p = 10^−5^, Fisher's exact test, Figure S9A). (*v*) Further, we expanded the TRN by adding 114 protein-DNA interactions discovered by a new bacterial one-hybrid reporter system termed TB1Hybrid [65], being that 31 interactions were exclusively identified by this method. (*vi*) Finally, we performed operon-based network expansion, propagating a TF's regulatory effect to all members of the operon containing a given TG. The final expanded *Mtb* TRN contained 1133 genes (28% of the genome) and 1801 regulatory links, more than a half of which were experimentally determined (Figure S9B, C); the complete list of interactions is available in the Table S12.

The global TRN provides a static summary of all possible regulatory interactions that mycobacteria may use when facing a broad spectrum of environments, ranging from normal to stressful conditions inside the Mφ. However, previous work has suggested that only parts of the network are utilized in specific conditions [36]. Such parts (subnetworks) function as network modules regulated by a hierarchy of transcription factors in an environment-dependent fashion. To understand how the surviving subset of *Mtb* bacilli specifically utilizes the TRN modules during prolonged intracellular infection, we analyzed the temporal response of the TRN by overlaying the 14-day Mφ infection time-course array data on the extended *Mtb* regulatory network.

We improved the earlier method called NetReSFun (Network Response to Step Functions), which identifies responsive TF-regulated subnetworks from time course microarray data [36]. NetReSFun computes the *Cov-score* (Methods) to quantify the expression change within the module (subnetwork) that we define as the total genes directly regulated by a given TF. A significant *Cov-score* indicates subnetwork response, when the expression levels of the subnetwork's gene members are either down- or upregulated during consecutive time points (*t*, *t+1*). Alternatively, simultaneous change of a TF's direct target genes may also be a surrogate of the TF's activity. Under this assumption, NetReSFun may recognize TFs that are “turned on” through posttranslational modifications such as phosphorylation and metabolite binding, but may or may not show increased expression levels themselves.

**Figure 8:**
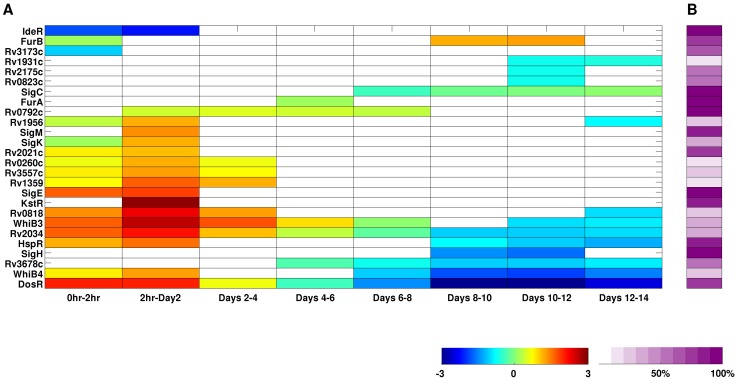
Temporal network response during macrophage infection. (A) Most of TF-controlled subnetworks represented in the TRN exhibit peaks of upregulation early in the infection followed by a repressive phase in later time points. The color scale indicates the overall direction of the transcriptional change within the subnetwork, from positive (+3) to negative (−3). (B) Percentage of regulatory links within the subnetwork based on experimental evidence.

The temporal map of network responses (Figure 8A) depicts specific TF-regulated subnetworks responsive during the time course, at a significance level of 0.05. The color scale indicates whether the overall trend of expression change within the subnetwork was positive (red) or negative (blue) at a given time interval. Intermediate colors denote subnetworks involving both up- and downregulated genes. The accompanying heatmap in Figure 8B indicates the source of regulatory links within the subnetwork (darker colors corresponding to higher fractions of links based on experimental evidence).

Strikingly, the map reveals that the dynamic utilization of the TRN occurs in a defined pattern that can be mapped back to distinctive phases of intracellular growth. In the first 2 days p.i. - which corresponds to the stress phase in the growth curve of Figure 1A – we observed a high number of responsive TF-subnetworks (20 out of 83), mostly exhibiting increased expression of involved genes. Among these were DosR, HspR, KstR, members of the WhiB family (WhiB3, WhiB4), two-component response regulators (Rv0260c, Rv0818, RegX3), and alternative sigma factors (SigE, SigK, SigM). The sharp induction of a large number of subnetworks is reminiscent of the general Environmental Stress Response (ESR) in yeast [66] and in *Bacillus subtilis* [67].

In contrast, after ∼6 days inside Mφ, we observed a reciprocal scenario where the TRN reflects a significant downmodulation of target genes, many of which had been induced immediately after invasion. This pattern is especially evident for a number of stress-responsive subnetworks that shift into downregulation during the slow growth phase, including RegX3, HspR and DosR. This “repressive” transcription phase indicates that the surviving bacteria have either adapted to stress, or they reside in a less hostile niche. For example, the SigH-controlled subnetwork displays a strong negative response only late in the time course (∼8 days and onwards). As a global regulator, SigH modulates the transcription of SigE and SigB, as well as its own promoter. Although SigH is not required for growth in Mφ, mutants lacking *sigH* caused reduced immunopathology and lethality in mice [68], [69]. Alternatively, it is possible that these regulatory changes are associated with the surviving bacilli reprogramming their physiology to assume the slow growth phenotype observed from day 4 onwards.

Importantly, we observed responsive subnetworks throughout the entire time-course, which indicates their importance for establishing productive infection. The presence of sustained responders such as HspR and DosR can have two possible implications. First, the opposite trends in the early phase of the infection (primarily upregulation) and later phases of infection (primarily downregulation) may indicate that the stress to which these modules respond initially is ameliorated at later time points. Alternatively, sustained responders may be necessary both to counteract initial phagosomal stress during the early phase of infection as well as for driving the persistor phenotype encountered in later phases of infection. This is the case of DosR, which is crucial for maintaining redox balance and energy levels during transitions into and out of dormancy-like conditions that perturb aerobic respiration, electron transport, or menaquinone pools [70]. HspR, which activates a subset of the heat-shock general stress response upon Mφ invasion [71], is also necessary in the persistent phase since Δ*hspR* strains exhibited attenuated growth in the chronic infection [72].

Finally, TRN analyses revealed novel sustained responders that might be critical for *Mtb* adaptation within the intracellular compartment. For example, the Rv2034-controlled subnetwork (inferred from *C. glutamicum* orthology) contains multiple *fadE* homologs implicated in β-oxidation of fatty acids and redox homeostasis. Notably, Rv2034 was recently characterized as an activator of the *phoP* virulence regulator in mycobacteria [73], which makes this regulator an interesting candidate for follow-up studies.

By overlaying the genome-scale temporal expression data onto the TRN, we revealed the activation of additional regulons during macrophage survival not readily apparent in our supervised analyses. Thus, by leveraging the behavior of multiple TG as a readout of TF activity, TRN analysis of time-course microarray data further enhances the ability to detect adaptive changes in *Mtb* gene expression during productive infection of macrophages.

### Conclusions

The process of infection is extremely dynamic as both host and pathogen seek to respond to the stimuli that they sense at their interface. In the current study we applied multiple analytical tools to establish a link between the transcriptional responses and physiological states through which *Mtb* transitions on its way to the establishment of a productive infection in its host macrophage. This analysis revealed several unexpected findings. Firstly, the initial phase of infection is marked by rapid bacterial replication coupled with effective bacterial killing by the macrophage. This is a period of marked stress for *Mtb*, which is illustrated by the greatest transcriptional response with respect to both up-regulated and down-regulated genes. Subsequently, the rate of replication slows and the bacterial number appears constant or at equilibrium, during which period the expression of many genes returns closer to control levels, whilst the divergent level of expression of others is sustained. Finally, the bacterial numbers start to increase indicating that the rate of replication exceeds that of death. At this time there is a marked down-regulation of many of the genes linked to general stress supporting the contention that *Mtb* has entered into a productive phase of infection characterized by enhanced intracellular survival. This is an important functional framework on which to hang the transcriptional profiles to determine which responses and/or metabolic themes impact which phase of infection.

Our examination of dynamic alternations of gene expression across the *Mtb* TRN also highlights stress responses and survival mechanisms deployed during distinct phases of host interaction. Among other things, our results indicate adaptive changes in lipid and energy metabolism akin to those observed in various dormancy models. Based on this, it is tempting to speculate that early infection of resting macrophages may serve to prepare *Mtb* for conditions encountered within granulomas after the onset of the adaptive immune response. Detailed understanding of the sensory and regulatory pathways required for *Mtb* virulence remains rudimentary at best. This point is illustrated by the large number of “genes of unknown function” which are actively regulated during intracellular survival.

In the discussion, we have presented the observed transcriptomic changes as the result of all *Mtb* cells sensing and responding in concert to phagosomal cues in the intracellular environment. Clearly, the microarray profiles in this study capture an average behavior over time of a population of intracellular bacilli that, as we have shown, exist in distinct vacuolar niches and presumably metabolic states. It could be argued that the changes in gene expression represent the minority of *Mtb* that fail to block P-L fusion mounting dramatic stress responses. However, our data appear to be inconsistent with this view. For example, we know that *Mtb* in P-L encounter Fe-deplete conditions whereas the average behavior of Fe-responsive signature genes reflects an Fe-replete environment (data not shown). Alternatively, the apparent up- and down-regulation may be due to the selective enrichment of pre-existing phenotypic variant cells with randomly upregulated stress response subnetworks, similar to the cell-to-cell heterogeneity reported by Aldridge *et al.* [74]. The rapid killing observed immediately after macrophage entry supports this scenario, suggesting that a subpopulation of bacilli capable of surviving the bottleneck may be present prior to infection, due to random phenotypic variation within the microbial population. However, the ability to block the induction of an acid regulon following Mφ invasion by chemical manipulation of phagosomal pH [12] would suggest that *Mtb* are altering gene expression in response to host-derived cues.

Thus, our data suggest that the dominant transcriptional profiles highlighted here represent the adaptive responses of the majority population. Analysis at the single-cell level will be required to explore the strategies employed by *Mtb* to survive across diverse host environments experienced by each individual bacterium. To address this, we have begun to exploit fluorescent reporter strains responsive to specific environmental cues to gain a high-resolution view of *Mtb* intracellular adaptation and cell biology. Interestingly, the expression of an acid-inducible locus required for normal intracellular survival, *aprABC*, is expressed at distinct levels by individual bacilli within the same host cell [21]. The impact of this type of heterogeneity on *Mtb*-host interactions and pathogenesis has yet to be determined. Previous work has shown that stochastic variation (intrinsic to individual cells) can aid survival in stressful environmental conditions [75]. However, a similar role of extrinsic, environmental variation is yet to be established.

The identification of mutants attenuated for intracellular survival is a popular and powerful tool for defining *in vivo* survival mechanisms, however, there are some limitations to this methodology that can be addressed by transcriptional profiling. Array-based mutant screens such as Transposon Site Hybridization (TraSH) [76], [77] are in essence end-point assays that are not readily amenable to quantitative kinetic analyses. In addition, there are several well-characterized examples of genes known to be required for Mφ survival, such as *pckA* [42], [43], *icl* ([44], [78], *prpCD* [56], *katG* [79], [80], or *phoPR* [9], [81], that have not been identified by TraSH screens indicating that the method is not comprehensive [82]. The TraSH methodology may preferentially identify mutants with severe survival defects, while being less effective at isolating mutants with less extreme phenotypes, such as Δ*phoPR*, that resist killing but fail to grow within phagosomes [9]. Finally, mutant screens are limited by their inability to query the *in vivo* role of genes that are essential *in vitro* or identify genes whose phenotype upon inactivation is masked by compensatory changes in gene expression. Future studies would benefit from the coordinated application of these two distinct but complementary approaches to identify genes contributing to the pathogenesis of *Mtb*.

We feel that the significance of this current study is that it transforms transcriptional profiling from a purely descriptive analysis to the generation of a predictive discovery tool that can be used to identify genes, and therefore metabolic pathways and physiological states, that are required to support distinct phases in the intracellular life cycle of *M. tuberculosis*.

## Materials and Methods

### Bacterial Strains and Cells

As previously described [83], the *M. tuberculosis* clinical isolate strain CDC1551 [84], [85] was cultured in Middlebrook 7H9-OADC medium in ventilated T-75 flasks without shaking. Bone marrow-derived macrophages (Mφ) were isolated from C57BL/6 mice and grown in DMEM supplemented with 20% L-cell conditioned medium, 10% fetal calf serum (FCS), and antibiotics (penicillin and streptomycin). Media lacking antibiotics was added to Mφ at least 24 hr prior to infection with *M. tuberculosis*.

### Survival Assays

To monitor the survival/growth of *Mtb* in Mφ, confluent Mφ monolayers in 24-well dishes were infected (MOI ∼1∶1) with *Mtb* CDC1551. After 2 hr (t = 0) and at two day intervals up to 14 days, intracellular *Mtb* released from monolayers by lysis with ddH_2_O+0.05% Tween-80 were serially diluted, and plated on 7H10+cycloheximide agar. CFU were enumerated after ∼3 weeks incubation at 37°C. The overall integrity of Mφ monolayer was verified by microscopy.

### Replication Clock Plasmid

In order to measure the dynamics of *Mtb* replication and death during Mφ infection, a replication clock plasmid, pBP10, was used (generous gift of Dr. David R. Sherman). As previously described [15], in the absence of kanamycin selection pBP10 is lost at a rate proportional to the growth rate. Macrophage survival assays using *Mtb* CDC1551 harboring pBP10 (introduced by electroporation) were conducted as described above except that aliquots were plated on 7H10+cycloheximide agar both with and without 25 µg/ml kanamycin. Based on the loss of kanamycin resistance over time, rates of replication and death were quantified using the mathematical model of Gill *et al.* [15].

### Electron Microscopy

In parallel with Mφ-*Mtb* infections for survival assays and microarray analysis, confluent monolayers of Mφ were infected with *Mtb* CDC1551 at a low MOI (1∶1) as described above. At select times p.i. (2 hr and day 2-day 14, alternating days), samples were fixed in buffered glutaraldehyde solution (2.5% glutaraldehyde in 0.1 M sodium cacodylate, 5 mM CaCl_2_, 5 mM MgCl_2_, 0.1 M sucrose, pH 7.2), rinsed with 0.1M sodium cacodylate buffer (see above), post-fixed with 1% osmium tetroxide (4% stock osmium diluted in 0.1 M sodium cacodylate, 5 mM CaCl_2_, 5 mM MgCl_2_, pH 7.2), rinsed with buffer, soaked in 1% aqueous uranyl acetate, and then rinsed with water. Samples were then dehydrated in a graded ethanol and propylene oxide series, followed by gradual infiltration of Spurr's resin. Blocks were then polymerized and ultrathin sections (∼70 nm) were cut and contrasted with both lead citrate and uranyl acetate. In independent experiments to determine phagosome-lysosome fusion by the colocalization of *Mtb* with colloidal gold, at various time-points after infection (2 hr, day 2, 6, and 10) infected monolayers were pulsed with 15 nm colloidal gold (Aurion) for 2 hr, washed, and then chased for 45 min in infection media before fixation as above. In assessment of the morphology of intracellular *Mtb*, bacilli were considered intact if they maintained a rod shape (longitudinal sections) or circular shape (cross sections), if ultrastructural organization and electron opacity of the cytoplasm was preserved, and if no breaks in the cytoplasmic membrane or cell wall were detected. Otherwise, bacilli were counted as damaged.

### Macrophage Infection, RNA Isolation and Linear Amplification

The methodology for transcriptional profiling of intramacrophage *Mtb* including RNA isolation, linear amplification, and hybridization has been described previously [12], [83]. Briefly, C57BL/6 bone marrow-derived Mφ were infected (MOI ∼10∶1) with *M. tuberculosis* CDC1551 from 2 hr up to 14 days (2 day intervals). In controls, aliquots of the same bacterial samples were incubated in flasks without a Mφ monolayer for 2 hr. Addition of guanidine thiocyanate-based lysis buffer selectively lysed Mφ and stabilized bacterial RNA while leaving mycobacteria intact. Pelleted bacilli were lysed in 65°C Trizol using a BeadBeater and 0.1 mm silicon beads. Total RNA was isolated from Trizol lysates by chloroform extraction and Qiagen RNeasy column purification. To generate array targets, 250 ng of total RNA was amplified using the MessageAmp-II Bacteria RNA Amplification system (Ambion). Amino-allyl UTP was incorporated into aRNA during transcription to allow labeling with Alexa dyes.

### Target Preparation and Microarray Hybridization

Amino-allyl modified aRNA were labeled with Alexa Fluor 555 and Alexa Fluor 647 (Invitrogen) and purified using a MegaClear kit (Ambion). 10 µg of Alexa-labeled aRNA from paired samples was dried and resuspended in 75 µl of hybridization buffer (5× SSC, 25% formamide, 0.1% SDS, and 25 µg salmon sperm DNA). Slides were prehybridized for 1 hr in 25% formamide, 5× SSC, 0.1% SDS, 1% BSA and washed with H_2_O and isopropanol. Labeled targets were denatured at 95°C for 5 min, cooled to 60°C, and hybridized to microarrays at 45°C for 16–18 hr. Following hybridization, arrays were washed and processed for scanning as described previously [83]. The microarray platform used can be accessed via NCBI's Gene Expression Omnibus (GEO) database [86] under platform accession number GPL5754. This dataset has been deposited in GEO under series accession number GSE35362.

### Microarray Data Analysis

Microarrays were scanned with a GenePix 4000B instrument (Axon Instruments, Inc.) with preliminary image analysis, spot intensity determination, background measurements, spot quality assessment and flagging conducted using Imagene software (version 6.0, Biodiscovery). Poor quality spots with signal intensities less than three standard deviations above background were excluded from further analysis. Genes that were not flagged as Present in at least 14 of 16 slides were omitted from further analyses. Subsequent normalization, statistical analysis, and visualization of array data were performed with Genespring 7.3 (Agilent). We utilized the EDGE (*E*xtraction and analysis of *D*ifferential *G*ene *E*xpression) methodology of Storey et al. to identify time-dependent transcriptional changes [22], allowing detection of genes whose expression exhibited significant temporal trends but failed to meet static statistical cutoffs at any single time point. Genes with significant changes in expression levels relative to controls were identified based on both static (p<0.05 for at least one time point) and EDGE analysis (cubic spline of 4, q<0.03).

### Quantitative Real-Time RT-PCR (qRT-PCR)

RNA amplification and microarray methodology used in this study have been previously validated by qRT-PCR [12]. Additional qRT-PCR validation of temporal expression patterns of select genes during long-term Mφ infection was conducted by two-step real-time RT-PCR using iScript and iTaq SYBR Green reagents (Biorad). Each sample was analyzed in triplicate on an ABI 7500 starting with 100 ng of total RNA (amplified and unamplified). C_T_ values were normalized to values obtained for *sigA*, a constitutively expressed *Mtb* gene, and relative changes in gene expression were calculated using the 2^−ΔΔCT^ method [87].

### Subnetwork Response Identification Using NetReSFun

We modified the tool NetReSFun (89) in order to identify TF-regulated subnetworks sequentially responsive during TB lifespan in the macrophage environment. Consider a set of *t* step functions each of which “jumps” from 0 to 1 at time point τ:
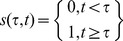
(1)NetReSFun compares gene expression profiles to these pre-defined step functions using scaled covariance to detect whether a gene's expression changed in the interval [τ-1, τ]. The method starts by computing the scaled covariance 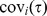 between the expression (log10-ratio) profile 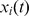 of gene *i* and a step function 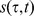 that jumps at time point τ:
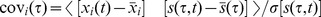
(2)where brackets denote averaging over genes, horizontal bar is averaging over time, and *σ* is the standard deviation. Thus, 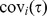 is the response of gene *i* during interval [τ−1, τ]. The combined response of subnetwork *I*, or *Cov-score*, is the mean of the absolute covariances of subnetwork genes
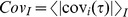
(3)To assess statistical significance, we compare the subnetwork response 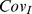 with a reference cumulative density function (c.d.f) 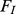, constructed with scores drawn of 1,000 random subnetworks (same size of subnetwork *I*, but assembled using nodes randomly chosen from the network). The density estimation of the c.d.f is done in a standard fashion by assigning probability mass 1/1000 for each observed random score; linear interpolation is applied to transform the discrete 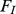 into a continuous function in the interval [0,1]. Finally, a p-value of observing 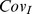 by chance can be readily estimated as below, and a p-value≤0.05 was considered significant.
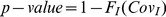
(4)To determine the direction of transcriptional change displayed in the temporal map (Figure 8), we first compute the deviation index of the subnetwork as the positive/negative ratio of individual covariances
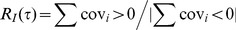
(5)The z-score of the deviation index, which relates the change of a particular subnetwork *I* to all other subnetworks, is given by
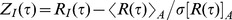
(6)where A denotes all TF-regulated subnetworks, brackets denote averaging over all subnetwork deviations, and σ is the standard deviation. Thus, the deviation z-score 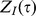 is the quantity plotted in the temporal map of Figure 8.

## Supporting Information

Figure S1 **Quantitative electron microscopy analysis of long-term** ***Mtb*** **-macrophage interactions.** At each timepoint, all visible intracellular *Mtb* in 100 macrophages were enumerated and categorized based on morphological criteria as described in Materials and Methods. (A) Morphology of intracellular *Mtb*. The proportion of intact, morphologically normal bacilli to damaged *Mtb* remained relatively constant over the 14-day infection. (B) Change in distribution of *Mtb* burden over time. The number of *Mtb* per macrophage increases steadily over time. Note, the large set of cells in which no *Mtb* were detected at early time points likely reflects the absence of detectable bacilli within the plane of section rather than uninfected cells.
(TIF)

Figure S2 ***Mtb*** **occupies heterogeneous intracellular niches during macrophage infection.** (A) Electron microscopy image shows dividing bacilli in “tight” vacuole, day 10 p.i. (B) Morphologically intact *Mtb* in spacious lysosome-like compartments surrounded by granular debris at day 6 p.i. This suggests *Mtb* can survive at least for a time in vacuoles that have fused with lysosomes. (C) *Mtb* surrounded by double-membrane (arrow) vacuole containing colloidal gold, consistent with an autophagosome (day 14 p.i.).
(TIF)

Figure S3 **Temporal expression profiling reveals novel genes differentially regulated within macrophage phagosomes.** (A) Gene tree (Euclidean distance measure) of 137 genes significantly upregulated at 48 hr p.i. novel to this study (compared to expression profiles at same time point reported in [26]). (B) Differential expression of “MT genes” during long-term macrophage infection. This geneset includes 292 predicted ORFs annotated in strain CDC1551 genome [88] not originally annotated in H37Rv genome [89] significantly regulated during long-term macrophage infection.
(TIF)

Figure S4 **Long-term intracellular expression of the core transcriptome.** Gene tree showing the distinct expression profiles of 215 genes comprising a core intracellular transcriptome previously defined based on their conserved presence and induction across a diverse panel of *Mtb* clinical isolates at 24 hr p.i. of resting macrophages [13].
(TIF)

Figure S5 **Genes of the Enduring Hypoxic Response (EHR) exhibit distinct expression patterns during long-term adaptation within macrophages.** Gene tree showing the transcriptional patterns of genes comprising the EHR, as identified by Rustad *et al.* [37], during long-term macrophage infection.
(TIF)

Figure S6 **Selective up-regulation of members of the KstR-dependent cholesterol regulon during intracellular growth.** (A) Gene tree showing the relative transcript levels of genes proposed to be directly controlled by KstR (Kendall *et al.* [51]) during long-term adaptation within macrophage phagosomes. Expanded views of distinct gene clusters are shown in (B–E). (B) Genes with early induction to moderate levels, most sustained throughout. (C) Genes with early induction to higher levels, with decrease at ∼day12 p.i., (D) KstR-dependent genes minimally responsive to phagosomal cues. (E) Genes displaying sustained, high level induction.
(TIF)

Figure S7 **Regulation of cell wall synthesis and composition in response to phagosomal cues.** Gene trees showing the sustained downregulation of genes involved in mycolic acid synthesis (A) and pthiocerol dimycocerosate (PDIM) (B).
(TIF)

Figure S8 **Regulation of ESX secretion systems during intracellular growth.** (A,B) Early induction of the ESX-1 secretion system including the main ESX-1 locus (A) as well as the EspR regulator and accessory factors Rv3614c-3616c encoded outside the RD-1 locus (B). These gene products function coordinately to facilitate secretion of ESAT-6/CFP-10 complex. [90], [91].
(TIF)

Figure S9 **The enlarged** ***Mtb*** **Transcriptional Regulatory Network (TRN).** (A) Number of regulatory links from each data source and corresponding overlaps. Links with experimental evidence originate from literature, as well as from the MtbRegList database and the TB1H assay. (B) Distribution of interactions based on their inference method. (C) Overview of the TRN, depicting protein-DNA interactions as edges linking TFs (blue triangles) to TGs (yellow circles).
(TIF)

Table S1 **Genes with peak induction at Day 2 followed by gradual decline toward control levels.**
(XLSX)

Table S2 **Genes significantly upregulated (>1.5X) at 48 hr novel to this study (compared to expression of >1.5X in both amplicon and oligo array data reported by Schnappinger** ***et. al (ref 29)***.
(XLSX)

Table S3 **Temporal expression profile of “MT genes”, predicted ORFs annotated in strain CDC1551 genome not originally annotated in the H37Rv genome.** Note, the gene order does not match the accompanying gene tree in Fig. S3B.
(XLSX)

Table S4 **Figure 5A - G** **enes with Early, transient induced temporal profile. **
(XLSX)

Table S5 **Figure 5B (red) - Genes with Early Induced, Sustained temporal profile.**
(XLSX)

Table S6 **Subsets of previously identified core intracellular transcriptome display distinct temporal expression profiles during 14-day macrophage infection.**
(XLSX)

Table S7 **Figure 5B** **(black) - Genes with Early Repressed, Sustained temporal profile.**
(XLSX)

Table S8 **Figure 5C -G** **enes with Steady, sustained induced temporal profile.**
(XLSX)

Table S9 **Figure 5D -G** **enes with Steady, sustained repression temporal profile.**
(XLSX)

Table S10 **Figure 5E - G** **enes with Delayed induced temporal profile.**
(XLSX)

Table S11 **Figure 6B - G** **enes with** ***hspX*** **-like temporal profiles.**
(XLSX)

Table S12 ***M. tuberculosis*** **Expanded Transcription Regulatory Network (TRN).**
(XLSX)
