# Supplementary figures and images for: Linking the Transcriptional Profiles and the Physiological States of Mycobacterium tuberculosis during an Extended Intracellular Infection
Source: PLoS Pathog. 2012 Jun 21;8(6):e1002769. doi: 10.1371/journal.ppat.1002769 (PMC3380936; doi:10.1371/journal.ppat.1002769)

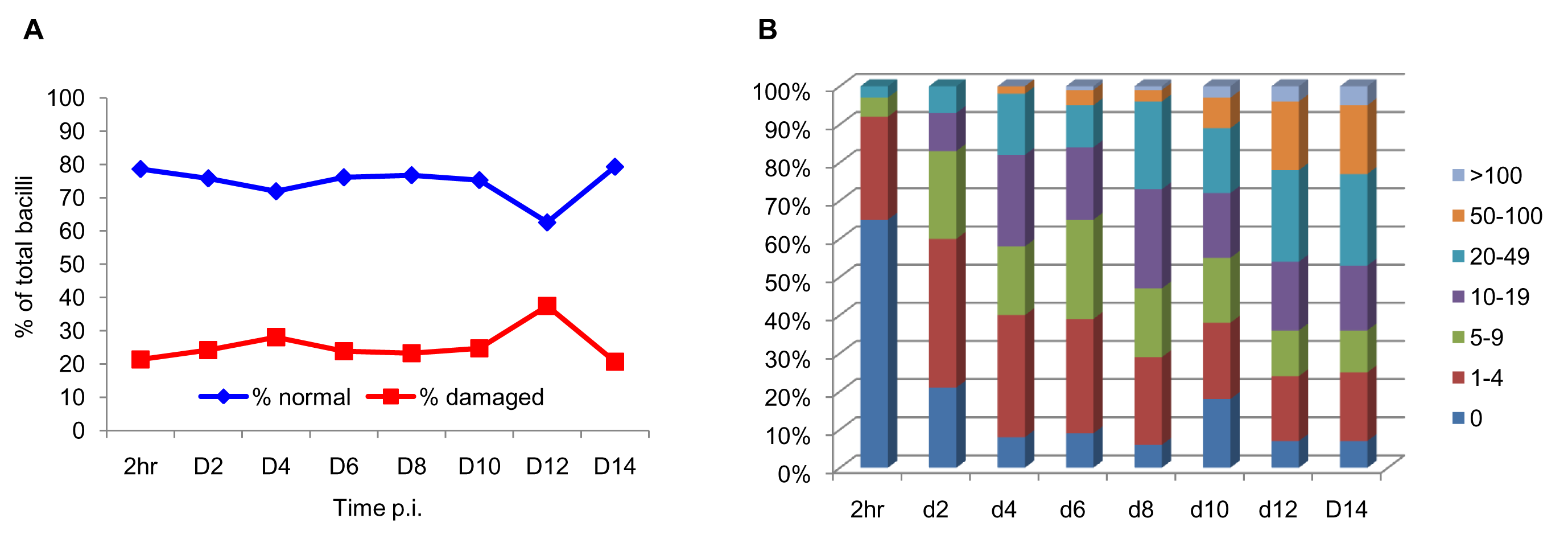

Supplement: Figure S1 — Quantitative electron microscopy analysis of long-term Mtb -macrophage interactions. At each timepoint, all visible intracellular Mtb in 100 macrophages were enumerated and categorized based on morphological criteria as described in Materials and Methods. (A) Morphology of intracellular Mtb. The proportion of intact, morphologically normal bacilli to damaged Mtb remained relatively constant over the 14-day infection. (B) Change in distribution of Mtb burden over time. The number of Mtb per macrophage increases steadily over time. Note, the large set of cells in which no Mtb were detected at early time points likely reflects the absence of detectable bacilli within the plane of section rather than uninfected cells. (TIF) [file ppat.1002769.s001.tif]

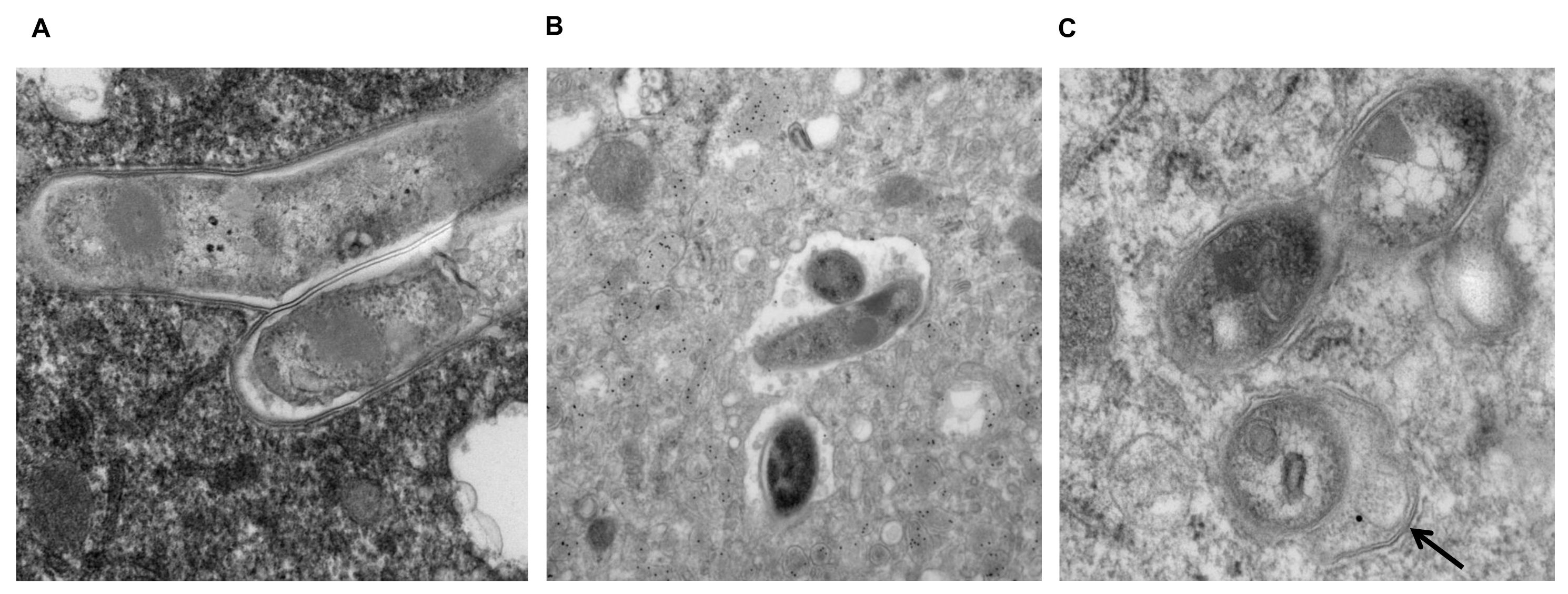

Supplement: Figure S2 — Mtb occupies heterogeneous intracellular niches during macrophage infection. (A) Electron microscopy image shows dividing bacilli in “tight” vacuole, day 10 p.i. (B) Morphologically intact Mtb in spacious lysosome-like compartments surrounded by granular debris at day 6 p.i. This suggests Mtb can survive at least for a time in vacuoles that have fused with lysosomes. (C) Mtb surrounded by double-membrane (arrow) vacuole containing colloidal gold, consistent with an autophagosome (day 14 p.i.). (TIF) [file ppat.1002769.s002.tif]

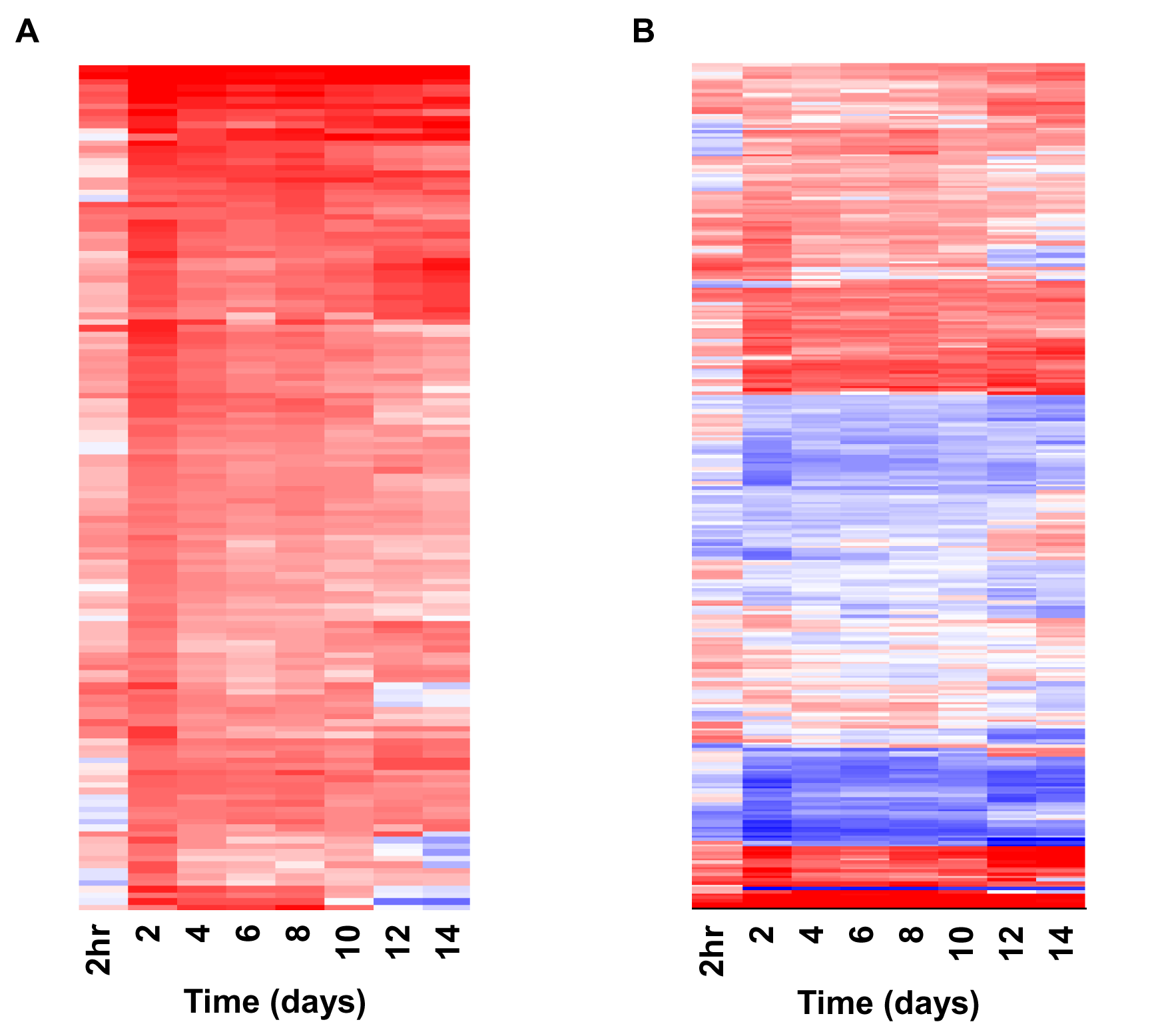

Supplement: Figure S3 — Temporal expression profiling reveals novel genes differentially regulated within macrophage phagosomes. (A) Gene tree (Euclidean distance measure) of 137 genes significantly upregulated at 48 hr p.i. novel to this study (compared to expression profiles at same time point reported in [26]). (B) Differential expression of “MT genes” during long-term macrophage infection. This geneset includes 292 predicted ORFs annotated in strain CDC1551 genome [88] not originally annotated in H37Rv genome [89] significantly regulated during long-term macrophage infection. (TIF) [file ppat.1002769.s003.tif]

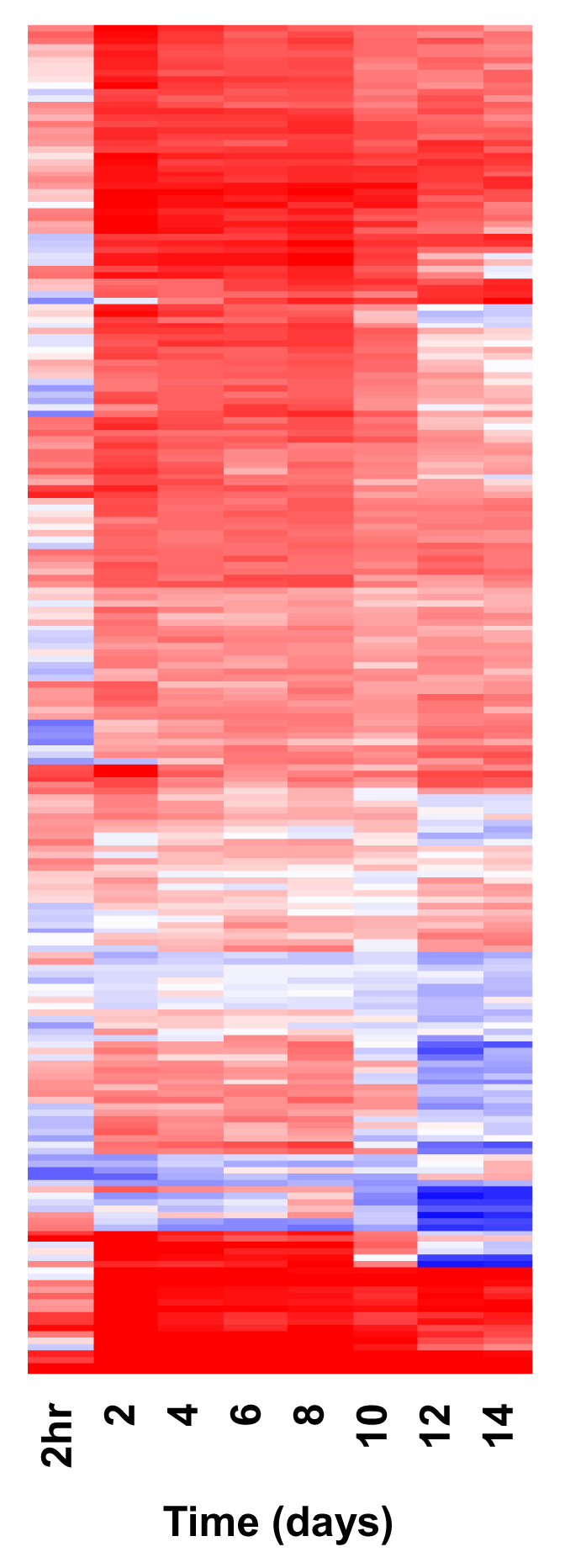

Supplement: Figure S4 — Long-term intracellular expression of the core transcriptome. Gene tree showing the distinct expression profiles of 215 genes comprising a core intracellular transcriptome previously defined based on their conserved presence and induction across a diverse panel of Mtb clinical isolates at 24 hr p.i. of resting macrophages [13]. (TIF) [file ppat.1002769.s004.tif]

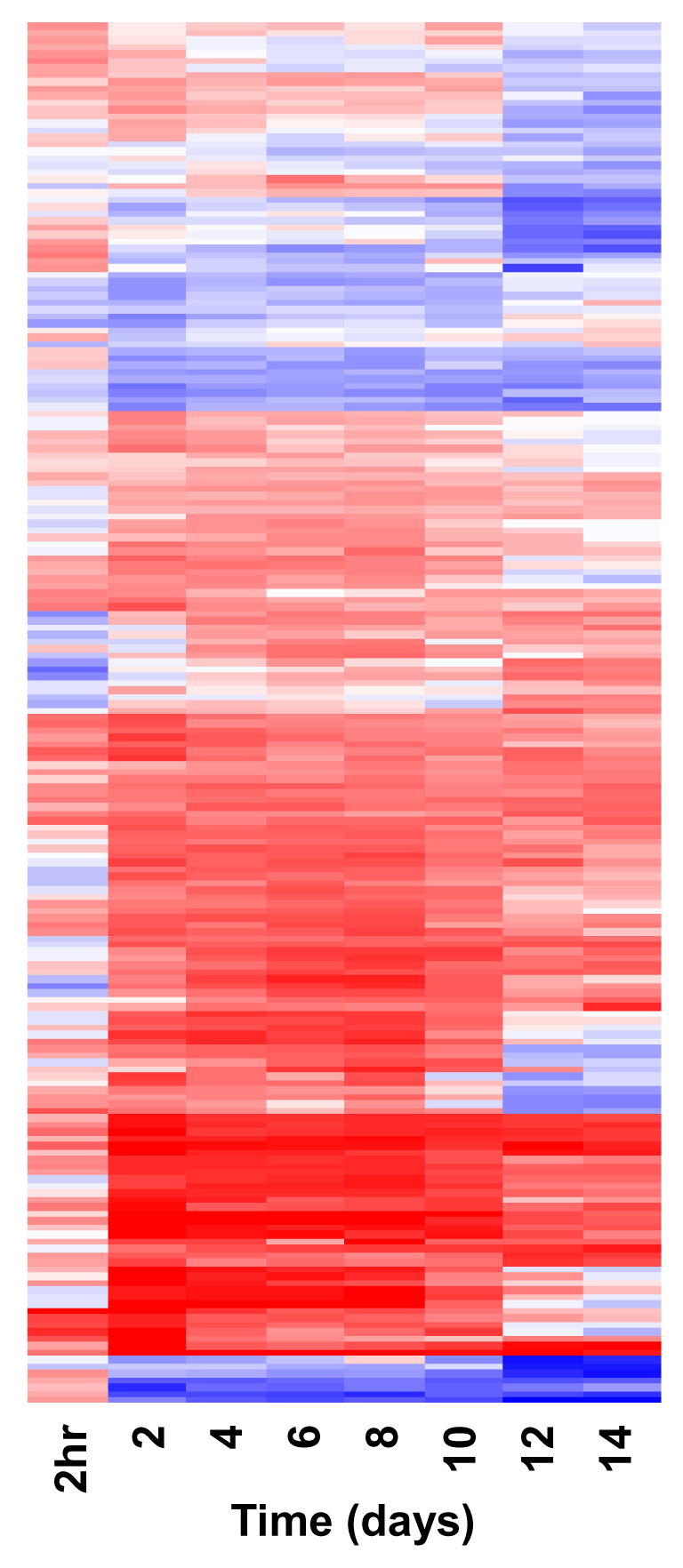

Supplement: Figure S5 — Genes of the Enduring Hypoxic Response (EHR) exhibit distinct expression patterns during long-term adaptation within macrophages. Gene tree showing the transcriptional patterns of genes comprising the EHR, as identified by Rustad et al. [37], during long-term macrophage infection. (TIF) [file ppat.1002769.s005.tif]

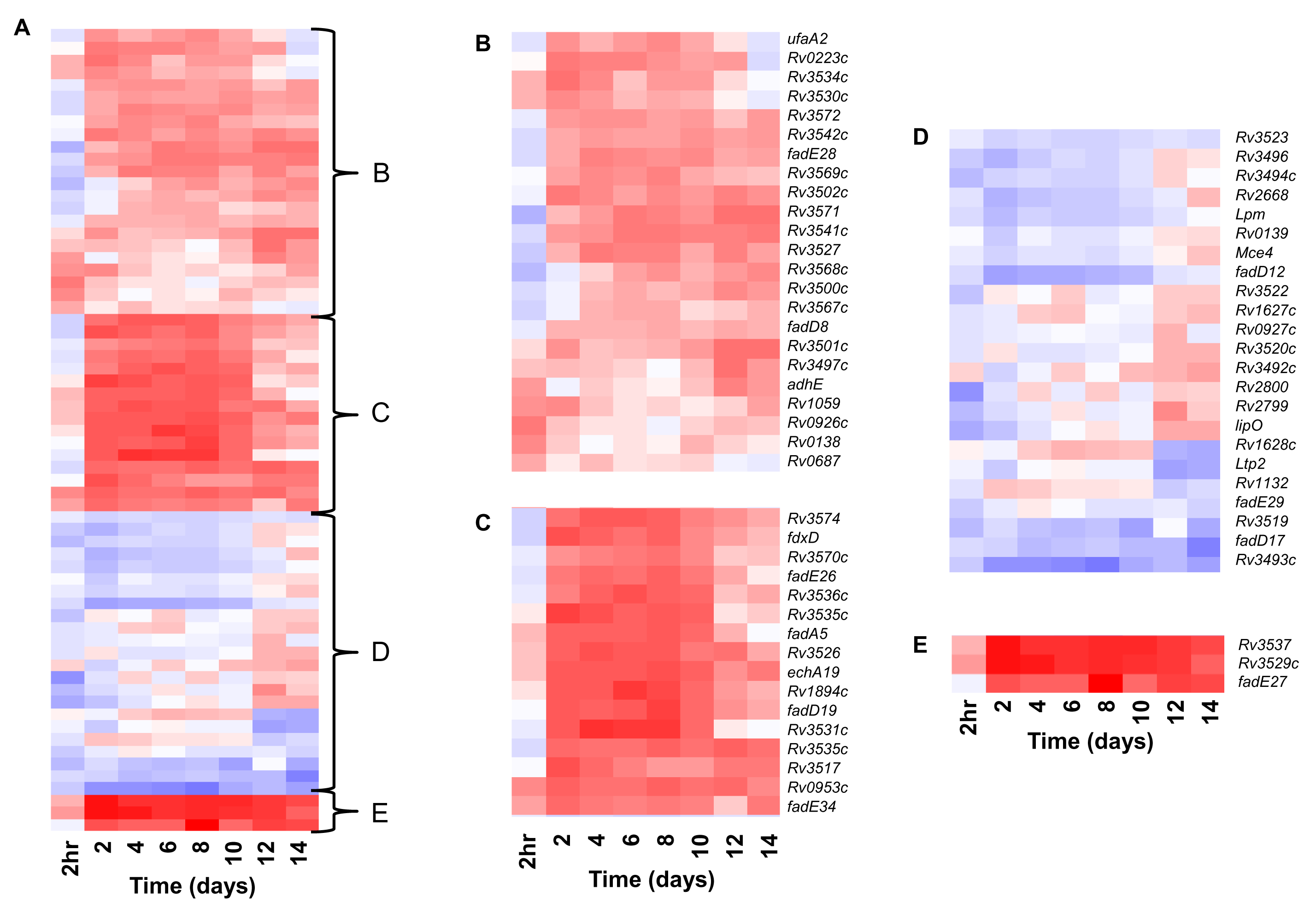

Supplement: Figure S6 — Selective up-regulation of members of the KstR-dependent cholesterol regulon during intracellular growth. (A) Gene tree showing the relative transcript levels of genes proposed to be directly controlled by KstR (Kendall et al. [51]) during long-term adaptation within macrophage phagosomes. Expanded views of distinct gene clusters are shown in (B–E). (B) Genes with early induction to moderate levels, most sustained throughout. (C) Genes with early induction to higher levels, with decrease at ∼day12 p.i., (D) KstR-dependent genes minimally responsive to phagosomal cues. (E) Genes displaying sustained, high level induction. (TIF) [file ppat.1002769.s006.tif]

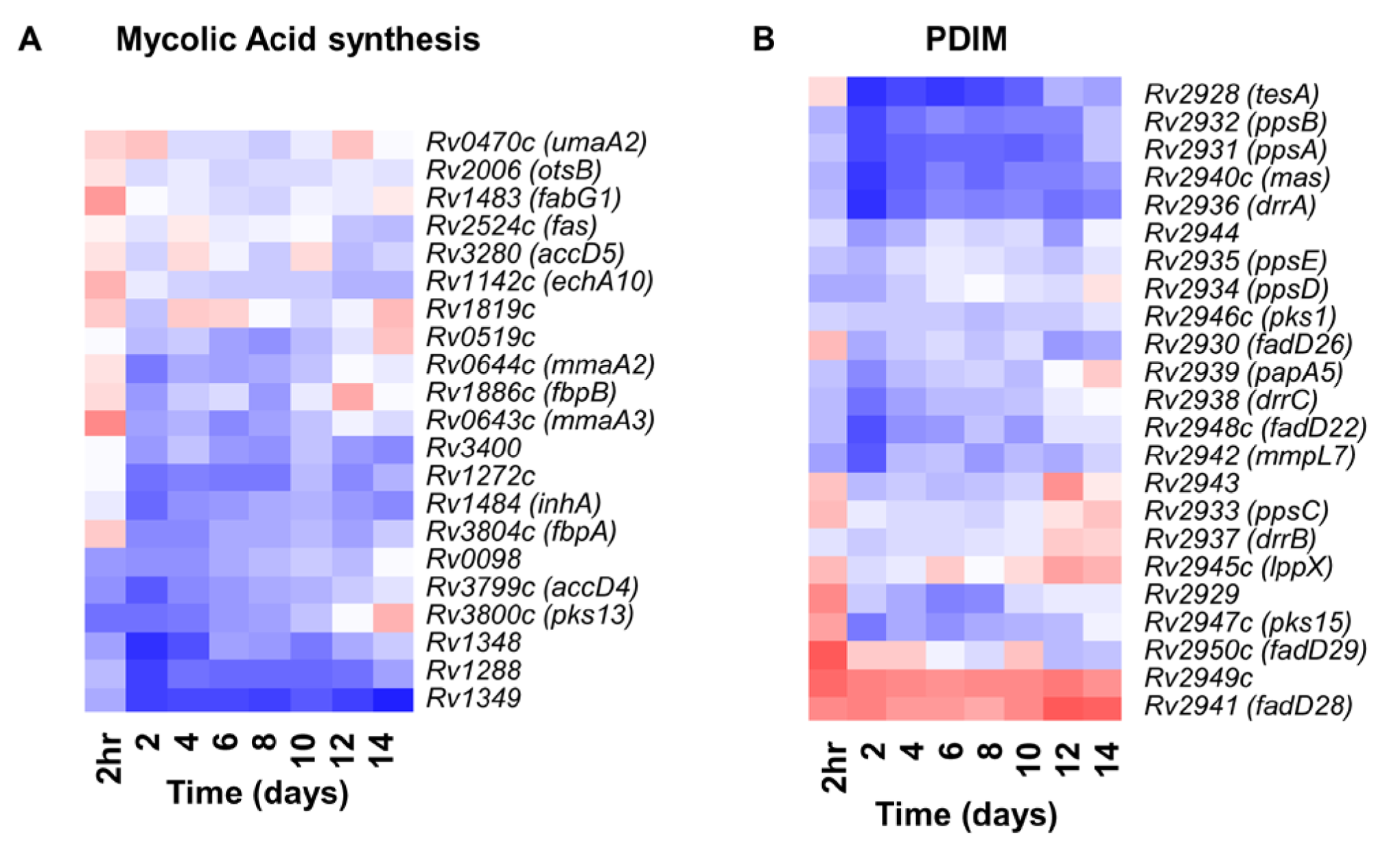

Supplement: Figure S7 — Regulation of cell wall synthesis and composition in response to phagosomal cues. Gene trees showing the sustained downregulation of genes involved in mycolic acid synthesis (A) and pthiocerol dimycocerosate (PDIM) (B). (TIF) [file ppat.1002769.s007.tif]

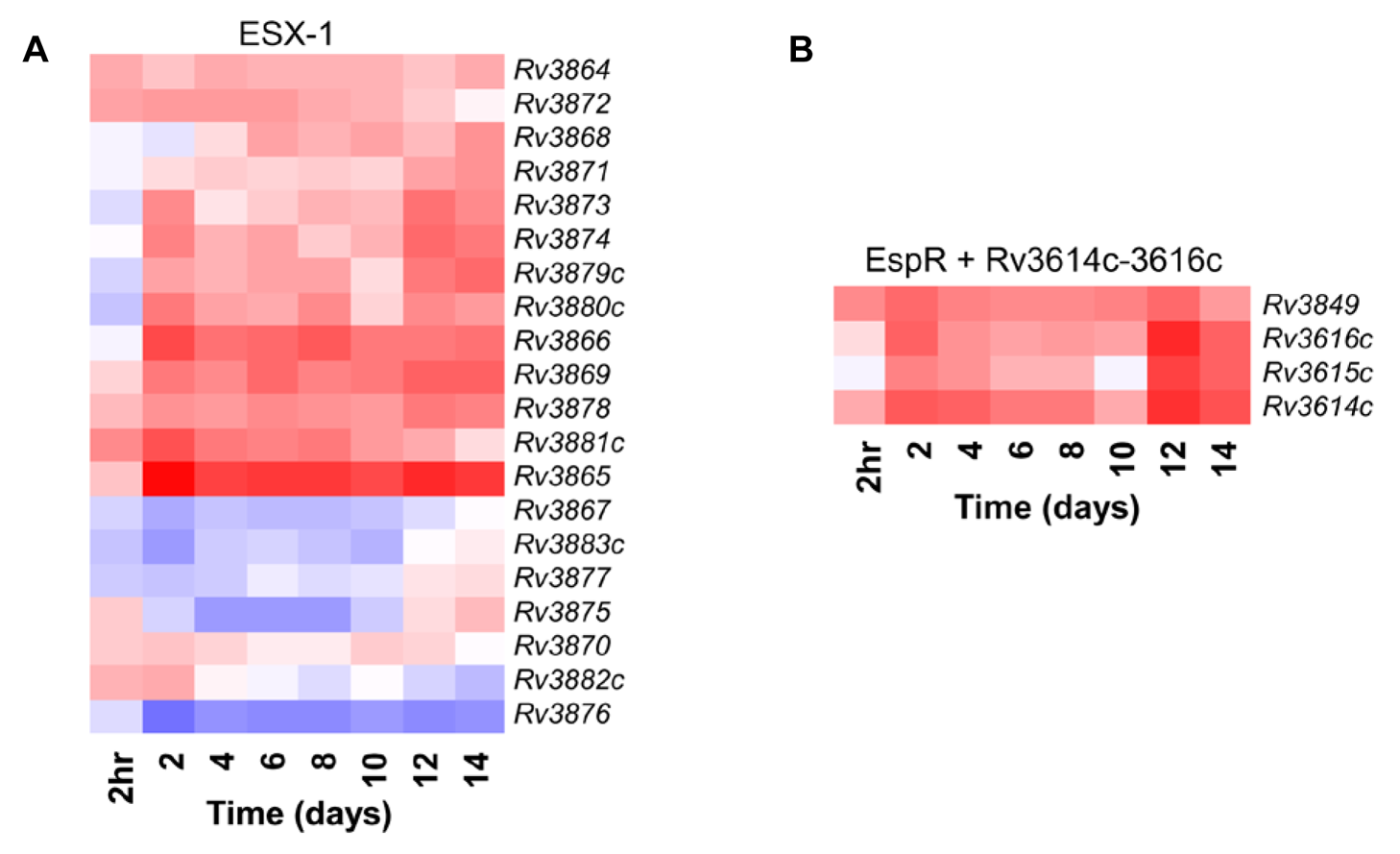

Supplement: Figure S8 — Regulation of ESX secretion systems during intracellular growth. (A,B) Early induction of the ESX-1 secretion system including the main ESX-1 locus (A) as well as the EspR regulator and accessory factors Rv3614c-3616c encoded outside the RD-1 locus (B). These gene products function coordinately to facilitate secretion of ESAT-6/CFP-10 complex. [90], [91]. (TIF) [file ppat.1002769.s008.tif]

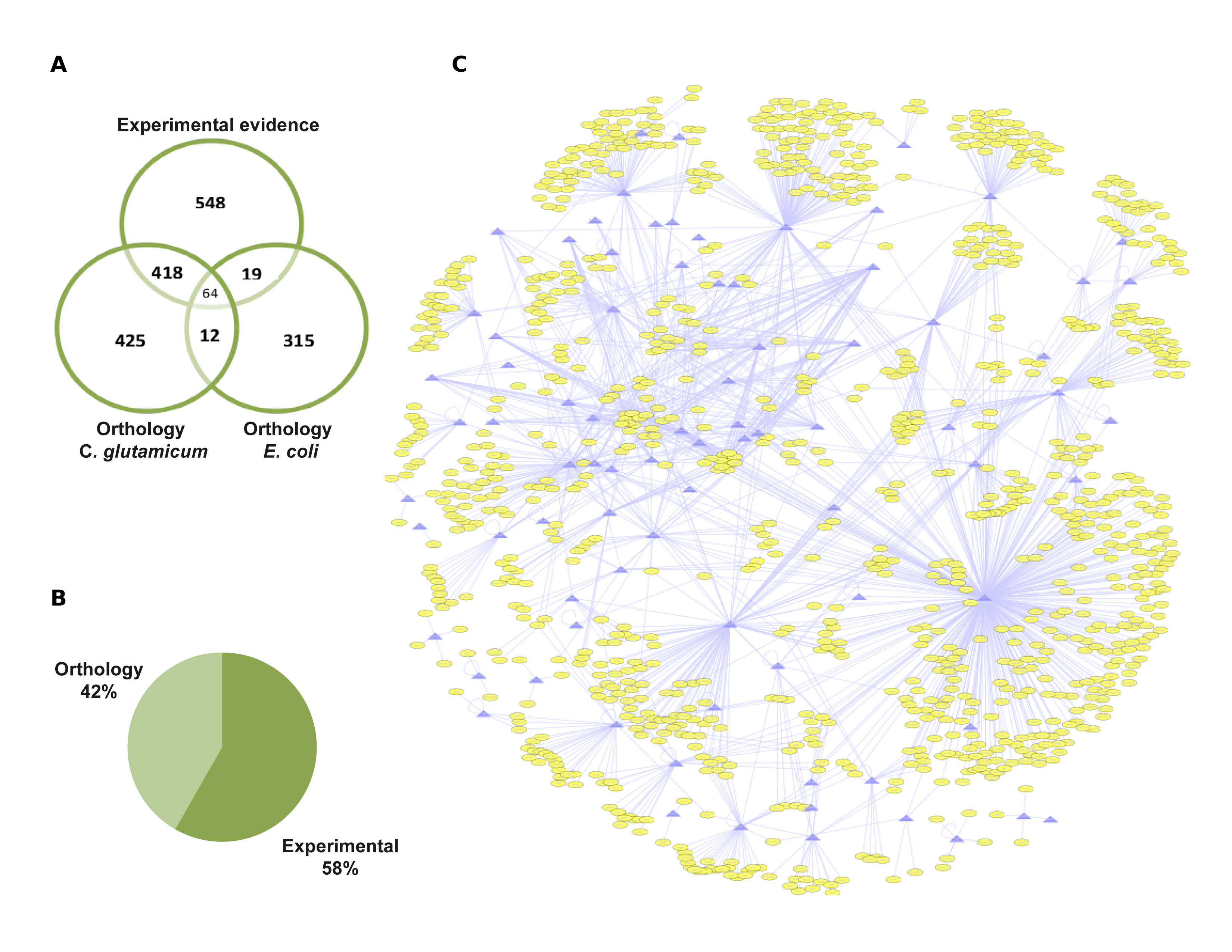

Supplement: Figure S9 — The enlarged Mtb Transcriptional Regulatory Network (TRN). (A) Number of regulatory links from each data source and corresponding overlaps. Links with experimental evidence originate from literature, as well as from the MtbRegList database and the TB1H assay. (B) Distribution of interactions based on their inference method. (C) Overview of the TRN, depicting protein-DNA interactions as edges linking TFs (blue triangles) to TGs (yellow circles). (TIF) [file ppat.1002769.s009.tif]
